# MYC-dependent recruitment of RUNX1 and GATA2 on the SET oncogene promoter enhances PP2A inactivation in acute myeloid leukemia

**DOI:** 10.18632/oncotarget.9840

**Published:** 2016-06-06

**Authors:** Raffaella Pippa, Ana Dominguez, Raquel Malumbres, Akinori Endo, Elena Arriazu, Nerea Marcotegui, Elizabeth Guruceaga, María D. Odero

**Affiliations:** ^1^ Hematology/Oncology Program, Center for Applied Medical Research (CIMA), University of Navarra, Pamplona, Spain; ^2^ Centre for Gene Regulation and Expression, College of Life Sciences, University of Dundee, Dundee, UK; ^3^ Instituto de Investigación Sanitaria de Navarra (IdiSNA), Pamplona, Spain; ^4^ Department of Biochemistry and Genetics, University of Navarra, Pamplona, Spain

**Keywords:** SET, PP2A, AML, MYC, RUNX1

## Abstract

The SET (I2PP2A) oncoprotein is a potent inhibitor of protein phosphatase 2A (PP2A) that regulates many cell processes and important signaling pathways. Despite the importance of SET overexpression and its prognostic impact in both hematologic and solid tumors, little is known about the mechanisms involved in its transcriptional regulation. In this report, we define the minimal promoter region of the *SET* gene, and identify a novel multi-protein transcription complex, composed of MYC, SP1, RUNX1 and GATA2, which activates *SET* expression in AML. The role of MYC is crucial, since it increases the expression of the other three transcription factors of the complex, and supports their recruitment to the promoter of *SET*. These data shed light on a new regulatory mechanism in cancer, in addition to the already known PP2A-MYC and SET-PP2A. Besides, we show that there is a significant positive correlation between the expression of *SET* and *MYC*, *RUNX1*, and *GATA2* in AML patients, which further endorses our results. Altogether, this study opens new directions for understanding the mechanisms that lead to *SET* overexpression, and demonstrates that MYC, SP1, RUNX1 and GATA2 are key transcriptional regulators of *SET* expression in AML.

## INTRODUCTION

Acute myeloid leukemia (AML) results from multiple genetic and epigenetic alterations in hematopoietic stem cells, and is characterized by a differentiation blockade of the myeloid hematopoietic progenitor cells accompanied by uncontrolled proliferation [1]. Although major improvements have been achieved in the overall survival of adult cases ≤60 years, most of the patients are older than 60 years, and in this group only 5-15% are cured [2, 3]. Therefore, it is necessary to develop more effective treatment strategies for this disease.

PP2A, one of the main serine/threonine phosphatases in mammalian cells, is a tumor suppressor which regulates several essential functions and counteracts most of the kinase-driven intracellular signaling pathways [4]. Our group and others have demonstrated that overexpression of SET, a potent PP2A endogenous inhibitor, is a recurrent event that causes PP2A inactivation in hematological neoplasms [5–8]. The *SET* gene (also known as I2PP2A or TAF-1β; 9q34) encodes a multifunctional protein that mediates essential functions in cell cycle control [9], cell migration [10], apoptosis [11–13], differentiation [14], DNA repair [15], DNA replication [16], transcription and histone acetylation [17–20], as well as chromosome modeling [21, 22]. SET was first identified as an oncogene fused with the nucleoporin NUP214 (CAN) in acute undifferentiated leukemia [23], and soon after, it was described as a PP2A inhibitor [24]. SET binds directly to the PP2A catalytic subunit, impairing its tumor suppressor enzymatic activity [5, 24–26]. Recent studies have revealed how SET inhibition of PP2A depends on SET sub-cellular localization [10, 27]. In steady-state cells, SET levels are low and it localizes mainly in the nucleus through the interaction with importin alpha3/beta [28]. In dividing cells, SET expression increases and it accumulates in the cytoplasm [29]. The nucleus-cytoplasm shuttling of SET is controlled by the interaction with exportin CRM1 [30], and by the phosphorylation of serine 9 in one of the SET nuclear localization signals [10, 31, 32]. Interestingly, the anticancer activity of FTY720 and OP449, two recently discovered PP2A-activating drugs, depends on the interaction/sequestration of SET, pointing out the significance of this oncogene in AML [26, 33–35].

Nevertheless, despite the importance of SET, and the prognostic impact of SET overexpression in both solid and hematologic tumors [5, 7, 8, 36–39], little is known about the mechanisms involved in the transcriptional regulation of this oncogene. In this report, we study the promoter region of *SET* in order to investigate the mechanisms that lead to *SET* overexpression in AML. We determine its minimal functional promoter region, and demonstrate that MYC, SP1, RUNX1 and GATA2 form a multi-protein transcriptional complex that is involved in the transcriptional activation of *SET* in AML.

## RESULTS

### SET knockdown by shRNA and siRNA results in the re-establishment of PP2A activity and consequent inhibition of AKT and ERK cell proliferation pathways

**Figure 1:**
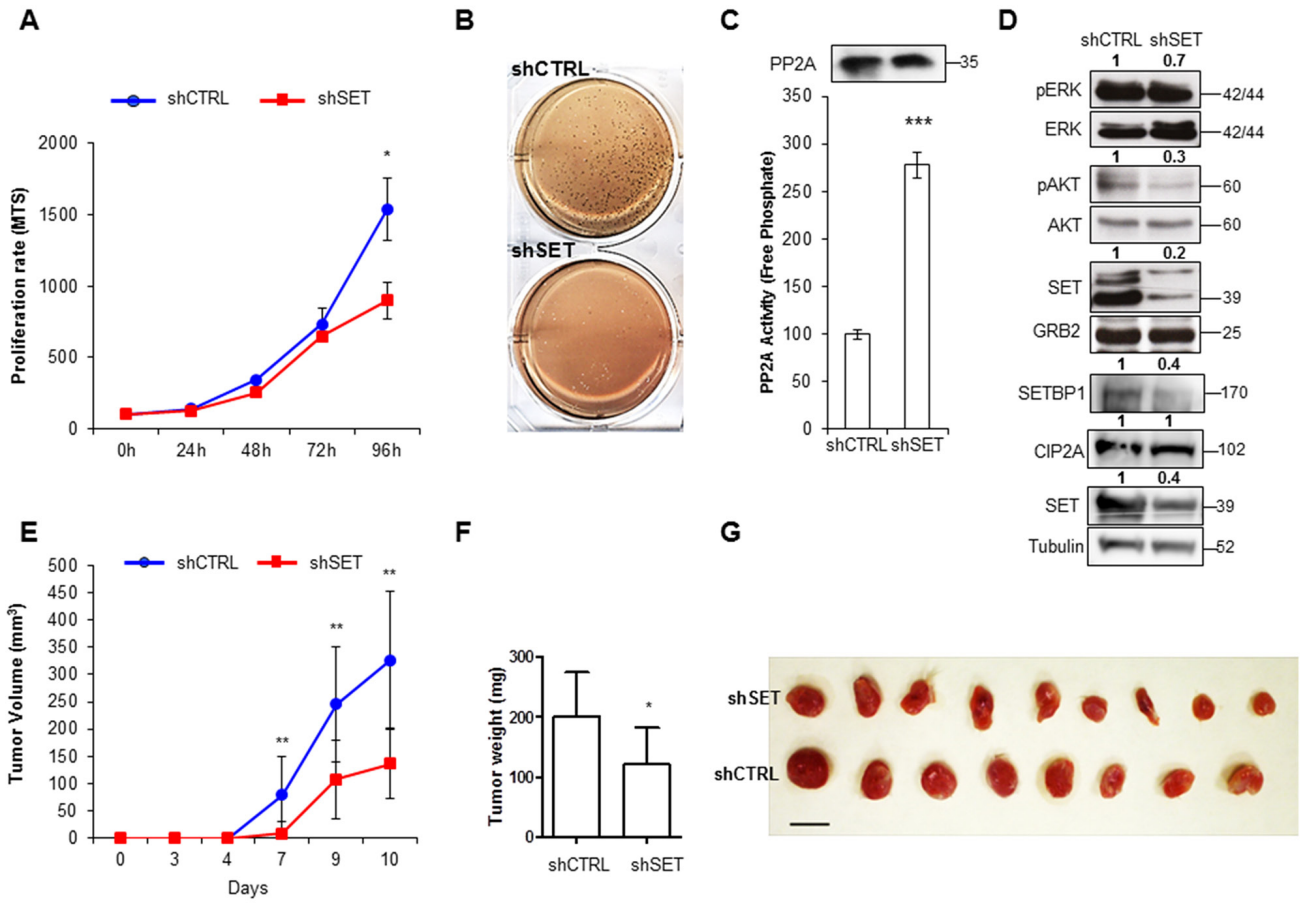
Depletion of SET results in the reduction of cell proliferation and clonogenic growth in AML **A**. Cell proliferation curve assessed by MTS assay in HL-60 cells with silenced SET shRNA (shSET) compared to control shRNA (shCTRL). **B**. Soft-agar clonogenic growth assay (colony forming capacity in soft-agar media), in HL-60 cells with shSET compared to control shRNA. **C**. PP2A activity assay with paired Western blot detection of the amount of PP2A immunoprecipitated. Data are the means ± SD of three independent experiments. **D**. Representative Western blot of the phosphorylation state of PP2A targets ERK (T202/Y204) and AKT (T308), CIP2A and SETBP1, together with the endogenous levels of SET, in cells stably transfected with shRNA of SET versus control. GRB2 and Tubulin were used as loading control. Numbers indicate the protein quantification relative to GRB2 or Tubulin and assessed using Image J software (NIH, USA). Tumor volumes **E**. and tumor weights **F**. in Rag2^-/-^ γc^-/-^ mice xenografts of HL-60 cells stably transfected with SET or control shRNAs, and the corresponding picture of the extracted tumors **G**.. Statistically significant differences are indicated: *P < 0.05, **P < 0.01, ***P < 0.001, Student's t-test analysis.

To explore the functional role of SET in AML, we transfected the AML cell lines HL-60 and HEL with specific shRNA and siRNAs that efficiently down-regulate SET levels. SET depletion led to a decrease in cell viability and clonogenic growth (Figure 1A and 1B, Supplementary Figure S1A, S1B, S1F, S1G), accompanied by an increase in apoptosis (Supplementary Figure S1C, S1H). Furthermore, PP2A activity was re-established, producing the inactivation of AKT and ERK, both targets of PP2A (Figure 1C, 1D and Supplementary Figure S1D, S1E, S1I, S1J). *In vivo* studies with mouse xenografts injected subcutaneously with SET shRNA-infected HL-60 cells produced tumors that grew at a slower rate (Figure 1E), and presented smaller and milder features compared to control shRNA cells (Figure 1F, 1G). To confirm that all these evidences were due exclusively to the depletion of *SET*, we analyzed the levels of CIP2A, another endogenous inhibitor of PP2A [40], and SETBP1, which is known to stabilize SET at protein level in AML [41]. No significant changes were found for CIP2A, whereas SETBP1 was significantly reduced (Figure 1D), suggesting a possible regulation of SETBP1 by SET. Taken together, these results show that SET depletion leads to a reduction in cell growth and an increase in apoptosis by re-activating PP2A in AML cells, confirming the importance of SET in AML.

### Identification and characterization of the *SET* minimal promoter region

As indicated above, *SET* is overexpressed in different solid and hematological tumors; however, the causes of this overexpression are still unknown. To address this issue, we investigated the functional promoter region of *SET*. First, we performed an *in silico* analysis of 1000 bp 5′ upstream of its TSS (transcription start site) for putative binding sites for transcription factors (TFs). This analysis revealed that RUNX1, GATA2, MYC and SP1, four TFs with essential roles in hematopoiesis [42–45], could have a role in the regulation of *SET*. The *SET* distal promoter region (-932/-699bp) contains DNA motifs for RUNX1 and GATA2, and the proximal promoter region (-318bp/TSS) for SP1 and MYC (Supplementary Figure S2). Upon assessing the expression of these TFs in six cell lines by Western blot (Supplementary Figure S3), we observed that all of them were present in AML cells, while non-AML cell lines lacked detectable RUNX1 expression, and showed low GATA2 expression.

**Figure 2:**
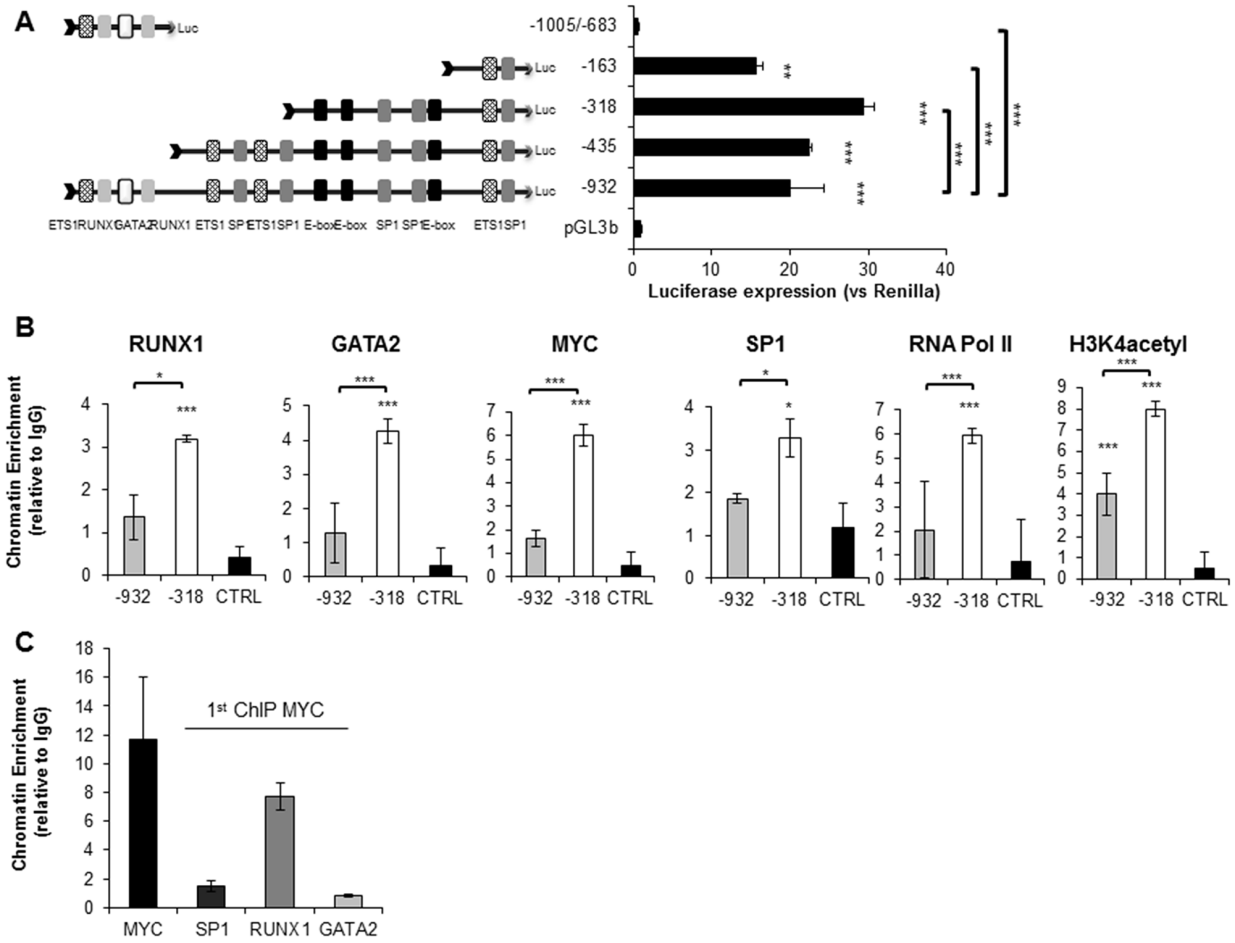
RUNX1, GATA2, SP1, MYC and RNA Pol II co-localize on the SET minimal functional promoter region (-318bp-TSS) Luciferase assays with the *SET* promoter constructs in HL-60 cells 48h after transfection. **A**. Results represent relative Firefly/Renilla luciferase activities considering the empty pGL3basic vector as 1. Data are the means ± SD of three independent experiments. Asterisks denote the statistical significance of the differences between pGL3b and the constructs or between the paired constructs where indicated. *P < 0.05, **P < 0.01, ***P < 0.001, Two-way ANOVA and Bonferroni tests were used. **B**. Chromatin Immunoprecipitation assay performed in the HL-60 cell line. Two promoter sub-regions were defined, a distal region (-932/-587bp) enriched with RUNX1 and GATA2 predicted binding sites and the minimal functional promoter region (-318/-144bp) with SP1 and MYC E-box putative binding sites. A distal genomic region on the same chromosome 9 was used as a negative control. QRT–PCR results were calculated using the 2-Ct method and they are presented as the fold enrichment of chromatin DNA precipitated by the specific antibody versus chromatin DNA precipitated by a non-related IgG. Values are the mean of three independent experiments. Asterisks denote the statistical significance differences where indicated. *P < 0.05, **P < 0.01, ***P < 0.001, Two-way ANOVA and Bonferroni tests were used. **C**. ChIP-re-ChIP assay performed in the HL-60 cell line. Technical procedures were carried out as described in Materials and Methods. MYC antibody was used for the first immunoprecipitation, and SP1, RUNX1, and GATA2 antibodies were used for the second immunoprecipitation. Re-ChIP assay values are the mean of two independent experiments.

Next, we analyzed this region using five different prediction algorithms in order to define the putative minimal promoter region (Supplementary Figure S4). These data allowed us to generate serial 5′ and 3′ truncation constructs that were cloned upstream of the luciferase reporter gene, and transfected into HL-60, HEL and HEK293t cells. Our results showed that the region between -318bp and the TSS encompassed the maximum promoter activity in the three cell lines analyzed (Figure 2A and Supplementary Figure S5A and S6). This is in agreement with the region most recurrently predicted as promoter by the different algorithms used for the *in silico* analysis. In HEL, the -932/TSS region presented weaker activity than in the other cells, suggesting the presence of a silencer element particularly active in this cell line. The activity of pGL3-1005/-683bp was almost absent, as in the case of the empty pGL3b vector. Sequence analysis of the -318/TSS region revealed the presence of three E-box sequences, which are potential binding sites for TFs such as MYC [46, 47]. To narrow down the region involved in the regulation of *SET*, we performed luciferase assays with sequence mutants that were defective in one, two, or all three E-box sequences. As displayed in Supplementary Figure S7, constructs defective for E-boxes n.1 and 2 reduce promoter activation by 20% in HL-60 and by 30% in HEL cell lines, implicating these E-box sequences as significant sites for TF binding and regulation of SET transcription. Taken together, these results show the importance of the -318bp/TSS region in the transcription of the *SET* gene.

Chromatin immunoprecipitation (ChIP) and re-ChIP assays confirmed the binding of MYC, together with SP1, RUNX1 and GATA2 to the -318/-144bp region of *SET* promoter in HL-60 and HEL cells, where they co-localize with RNA polymerase II (RNA pol II) (Figure 2B, 2C and Supplementary Figure S5B, S5C). Intriguingly, we found that RUNX1 and GATA2 bind more efficiently to the -318/-144bp region than to the distal promoter region, although there are no DNA motifs for these TFs in this region. Additionally, using antibodies that recognize histone acetylation (H3K4 acetylation), we confirmed that the -318bp/TSS is a transcriptionally active region in AML.

### MYC and SP1 enhance the expression of *SET* in AML

**Figure 3:**
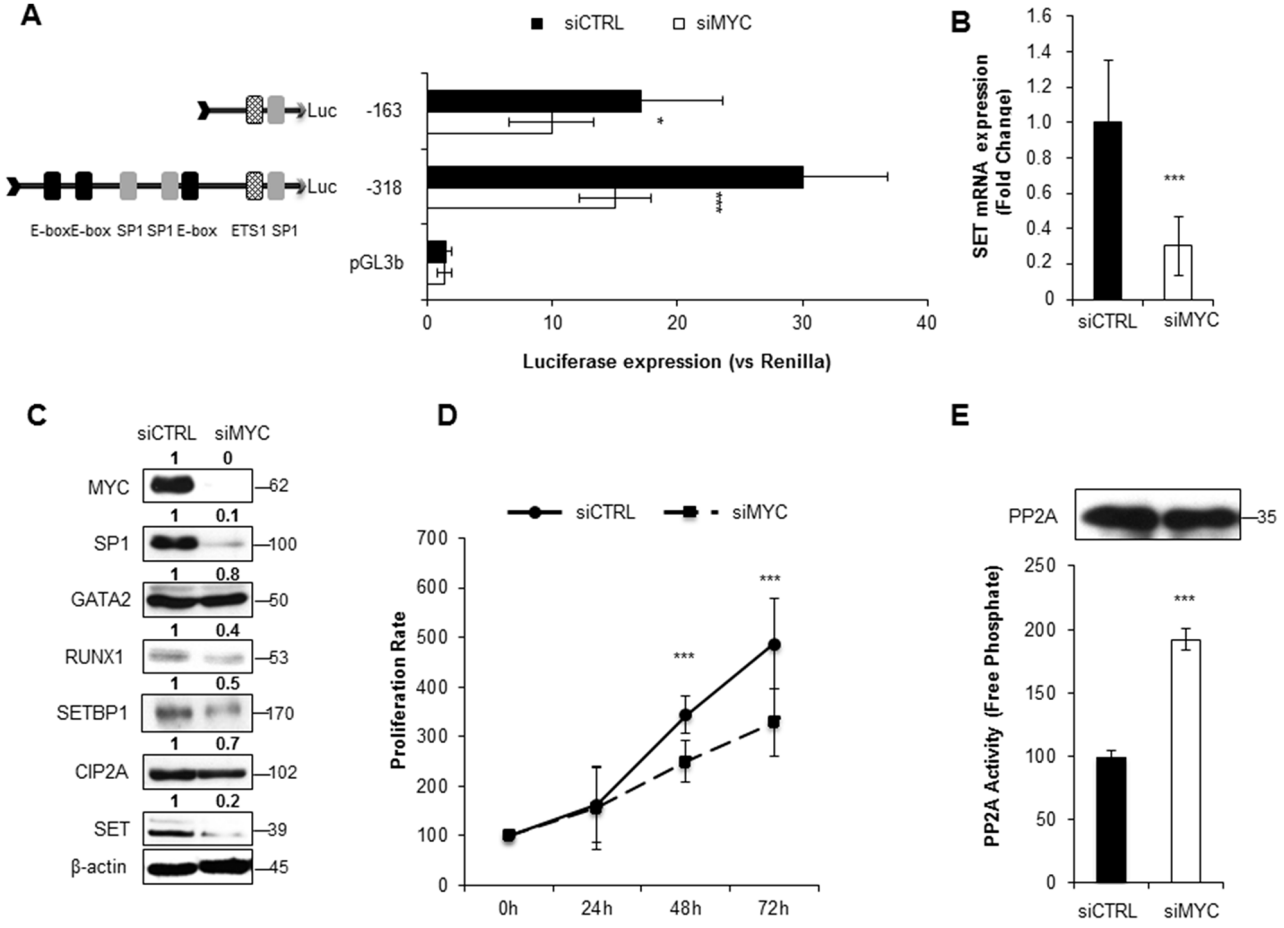
MYC depletion significantly reduces SET transcription and re-activates PP2A in AML **A**. Luciferase assay in HL-60 cells transfected with a siRNA for MYC silencing. **B**. *SET* mRNA expression assessed by QRT-PCR. **C**. Western blot analysis of the corresponding protein levels of MYC, SP1, GATA2, RUNX1, SETBP1, CIP2A and SET. β-Actin detection was used as loading control. Numbers indicate the protein quantification relative to β-Actin and assessed using Image J software (NIH, USA). **D**. Cell proliferation rates. **E**. PP2A activity levels with paired western blot results of the amount of PP2A immunoprecipitated in each condition. Data are the means ± SD of three independent experiments. *P < 0.05, **P < 0.01, ***P < 0.001, Students t-test analysis.

**Figure 4:**
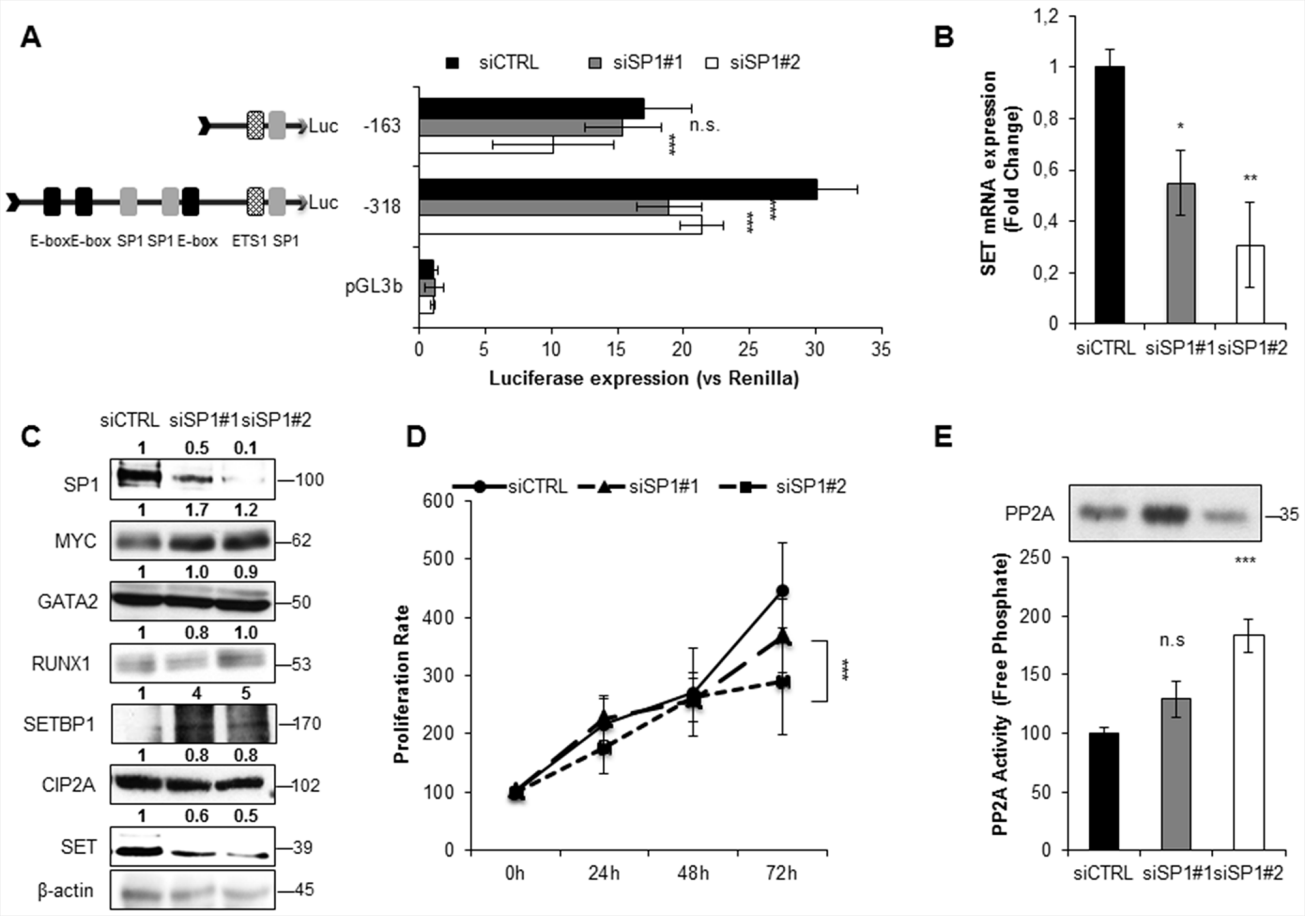
SP1 activates SET transcription in AML **A**. Luciferase assay in HL-60 cells transfected with two different siRNAs, siRNA#1 and siRNA#2. **B**. *SET* mRNA expression assessed by QRT-PCR. **C**. Western blot analysis of the corresponding protein levels of SP1, MYC, GATA2, RUNX1, SETBP1, CIP2A and SET. β-Actin was used as loading control. Numbers indicate the protein quantification relative to β-Actin and assessed using Image J software (NIH, USA). **D**. Cell proliferation rates. **E**. PP2A activity levels with paired western blot results of the amount of PP2A immunoprecipitated in each condition. Values are the mean ± SD of three independent experiments. *P < 0.05, **P < 0.01, ***P < 0.001, Students t-test analysis.

To investigate the role of MYC and SP1 in *SET* transcription, we silenced the expression of these two TFs with siRNAs. Luciferase assays showed a significant decrease in *SET* promoter activity, along with reduced SET expression at mRNA and protein levels (Figure 3A–3C, Figure 4A-4C and Supplementary Figures S8A-S8C and S9A-S9C). Remarkably, depletion of MYC caused a general reduction of all the other TFs involved in the transcription of *SET*, and also of the *SET*-related proteins CIP2A and SETBP1 (Figure 3C, and Supplementary Figure S8C). The SP1 reduction with SP1#1 siRNA had a similar effect, although in HEL cell line only (Supplementary Figure S9C). In addition, both SP1 and MYC knockdown decreased cell proliferation and re-activated PP2A (Figure 3D–3E, Figure 4D-4E, and Supplementary Figures S8D-S8E and S9D-S9E), indicating that these TFs activate the expression of SET, resulting in PP2A inactivation in AML cells. We also evaluated the effect of 10058F4, an inhibitor of MYC, on *SET* transcriptional regulation. Both SET promoter activity and levels (mRNA and protein) decreased markedly after treatment with 10058F4, mirroring the results obtained with the siRNA of MYC; furthermore, the PP2A function was re-established in HL-60 and HEL cells (Supplementary Figure S10 and S11). Finally, the inactivation of MYC produced a major decrease of SP1 (by 70%) and a more moderated reduction of GATA2 (by 20%) in HEL cells (Supplementary Figure S11C), whilst no significant effects on these TFs were found in HL-60 (Supplementary Figure S10C). These results confirm that MYC increases the expression of *SET* in AML, resulting in PP2A inactivation.

**Figure 5:**
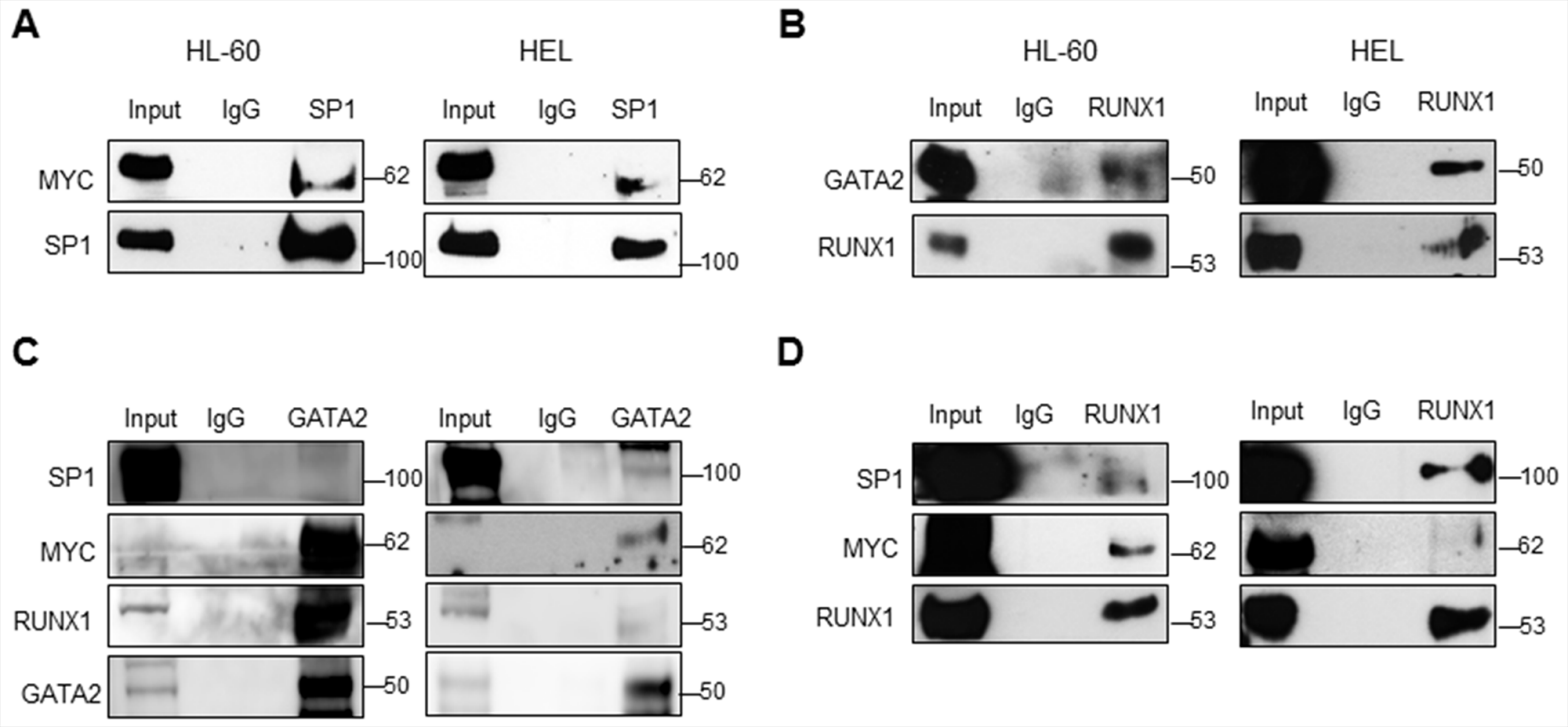
Transcriptional regulation complex that activates SET expression in AML Immunoprecipitation experiments in the HL-60 (left) and HEL (right) cell lines were performed using anti-SP1 or non-specific IgGs (used as a control). Then, the immunoprecipitates were analyzed for the presence of SP1 and MYC. **A**. Immunoprecipitation experiment using anti-RUNX1 followed by the analysis for the presence of RUNX1 and GATA2. **B**. Immunoprecipitation experiment using anti-GATA2 followed by the analysis for the presence of SP1, MYC, RUNX1 and GATA2. **C**. Immunoprecipitation experiment using anti-RUNX1 followed by the analysis for the presence of SP1, MYC and RUNX1. **D**. Input represents 30mg of total lysate. The western blots here displayed are the representation of three independent experiments.

SP1 is known to interact with MYC [48–50] and together they regulate the expression of several genes [51]. We therefore performed co-immunoprecipitation (co-IP) experiments and confirmed that SP1 binds to MYC in AML (Figure 5A). Altogether, our results demonstrate that MYC activates the expression of TFs such as SP1, forming a complex that enhances the transcription of *SET*, and eventually, the expression of CIP2A and SETBP1, resulting in PP2A inactivation and increasing cell proliferation in AML.

### MYC, SP1, RUNX1 and GATA2 form a multi-protein transcription complex that activates the expression of *SET*

**Figure 6:**
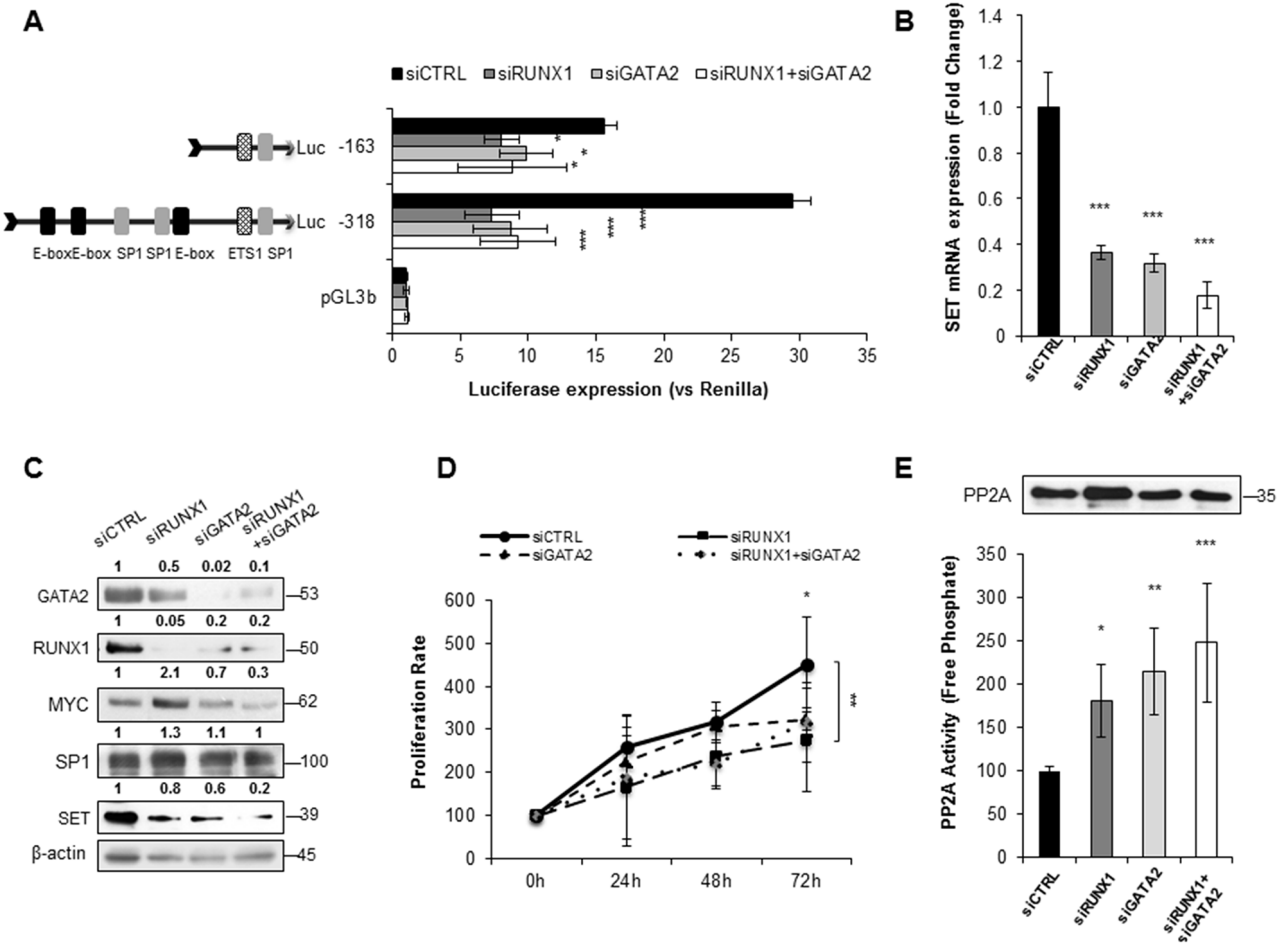
RUNX1 and GATA2 co-activate the expression of SET, and inactivate PP2A in AML **A**. Luciferase assay in HL-60 cells transfected with siRNAs for RUNX1 and GATA2, alone or together. **B**. *SET* mRNA expression analyzed by QRT-PCR. Expression was normalized to the *HPRT* housekeeping gene. **C**. Corresponding protein expression levels of GATA2, RUNX1, MYC, SP1 and SET were assessed by western blot. β-Actin was used as loading control. Numbers indicate the protein quantification relative to β-Actin and assessed using Image J software (NIH, USA). **D**. Cell proliferation of HL-60 cells transfected with siRNA for RUNX1, GATA2 and RUNX1 plus GATA2. **E**. PP2A activity levels with paired western blot analysis of the amount of PP2A immunoprecipitated in each condition. Values are the mean ± SD of three independent experiments. Asterisks indicate the statistical significance of the differences between the different constructs. *P < 0.05, **P < 0.01, ***P < 0.001, One-way ANOVA and Bonferroni post-hoc tests were used.

Since the ChIP and re-ChIP assays showed that RUNX1 and GATA2 bind to a region of the *SET* promoter that harbors predicted binding sites for SP1 and MYC, we performed immunoprecipitation experiments and confirmed the association between RUNX1 and GATA2, and their interaction with SP1 and MYC in HL-60 and HEL cells (Figure 5B–5D). Next, we further investigated the role of RUNX1 and GATA2 in the regulation of *SET* transcription. Knockdown of either RUNX1 or GATA2 markedly reduced *SET* promoter activity (mostly in the -318bp and -163bp regions), and mRNA and protein expression (Figure 6A–6C, Supplementary Figure S12A-S12C and Supplementary Figure S13). Moreover, as expected, PP2A activity was reconstituted and the cell proliferation rate decreased (Figure 6D, 6E and Supplementary Figure S12D, S12E). Single or simultaneous depletion of GATA2 and RUNX1 weakened the levels of MYC and SP1, revealing a complicated network of regulation among the TFs studied here that may vary depending on the cell line. In addition, overexpression of RUNX1 and GATA2 in HEK293t cells, which displayed undetectable and low levels of these TFs, respectively (Supplementary Figure S14), significantly increased the activity of the *SET* promoter and the amount of SET mRNA and protein, together with a gain in cell proliferation rate and PP2A inactivation (Supplementary Figure S14). No significant changes in *SET* expression were found when these two TFs were separately overexpressed (data not shown), suggesting that RUNX1 and GATA2 co-operation is necessary to activate *SET*. These data, together with the ChIP results, suggest the presence of a transcription complex that comprises at least MYC, SP1, RUNX1 and GATA2, which binds to the SET promoter and enhances its expression, along with the expression of each members of the aforementioned complex that may be the cause of PP2A inactivation in AML.

### MYC recruits RUNX1 and GATA2 and allows *SET* transcription in AML

**Figure 7:**
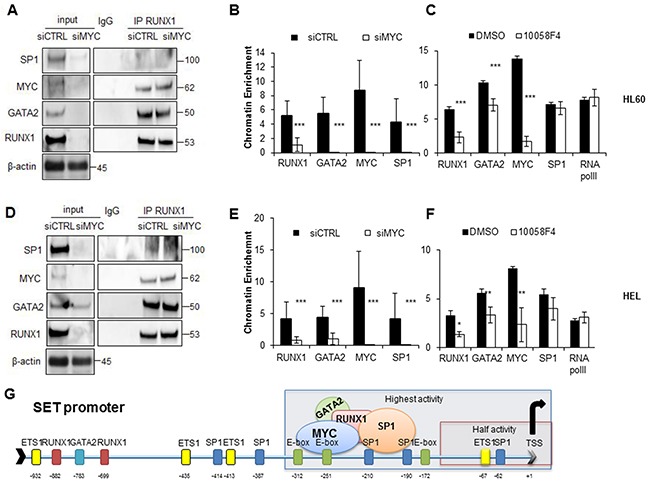
SET transcriptional regulation in AML is MYC-dependent **A**. Immunoprecipitation experiments using anti-RUNX1 or non-specific IgGs in HL-60 cells. Cell extracts 48h after the electroporation with siRNA against MYC or scramble siRNA followed by the analysis for the presence of SP1, MYC, GATA2 and RUNX1. β-Actin was used as loading control. **B**. ChIP assay performed in HL-60 cells with siRNA against MYC. **C**. ChIP assay performed in HL-60 cells treated with MYC inhibitor 10058F4. Values are the mean ± SD of three independent experiments. Statistically significant differences are indicated: *P < 0.05, **P < 0.01, ***P < 0.001, Mann-Whitney U test. The same experiments were performed in the HEL cell line **D, E, F. G** Model illustrating the participation of RUNX1, GATA2, MYC and SP1 in the activation of *SET* transcription in AML.

As the region of the *SET* promoter with maximum activity contains MYC binding sites, and these TFs interact among them, we studied whether depletion or inhibition of MYC could affect the formation and localization of the transcriptional complex of MYC, SP1, RUNX1 and GATA2. Co-IP and ChIP experiments in cells transfected with a siRNA for MYC show that the decrease of MYC, and consequentially reduction of RUNX1, GATA2 and SP1, did not totally prevent the formation of the multi-protein complex in the cells (Figure 7A, 7D). However, its localization on the *SET* functional promoter was entirely abolished (Figure 7B, 7E). Similar results were obtained in AML cell lines treated with the MYC inhibitor 10058F4. The inhibition and consequent reduction of MYC significantly reduced RUNX1 and GATA2 recruitment on the *SET* functional promoter (by 2.7 and 1.5 times *p* < 0.001, respectively in HL-60 cells (Figure 7C); and by 2.39 and 1.68 times *p* < 0.001 in the HEL cell line, (Figure 7F), whereas no substantial differences were found relative to SP1 or RNA pol II binding.

### *SET* expression correlates with *MYC*, *RUNX1* and *GATA2* expression in AML patients

**Figure 8:**
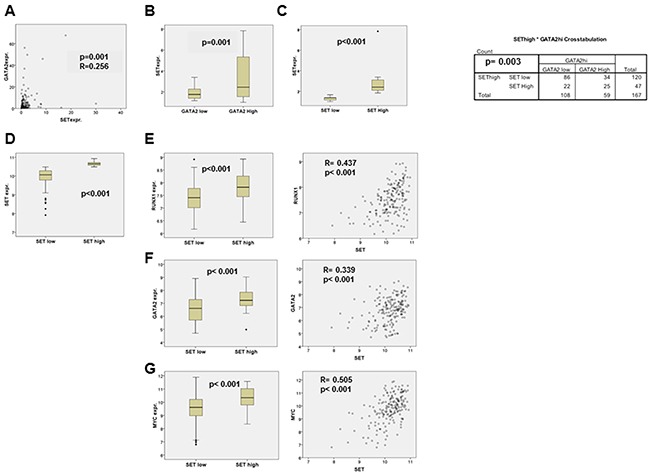
Correlation analysis of the SET and GATA2, RUNX1 and MYC expression in AML patients **A**. *SET* and *GATA2* mRNA expression levels in 167 AML patients assessed by QRT-PCR, and tested for correlation with SPSS 17.0 using Spearman's correlation test. The possible association between high/low *GATA2* and *SET* mRNA expression levels was tested by Pearson's chi square test, using the defined thresholds for *SET* and *GATA2* expression previously described [8, 52]. *SET* expression in the two groups of patients according to *GATA2* expression **B**. and in the two groups defined by *SET* expression levels **C**. are shown. Statistical analysis was performed with Mann-Whitney's U test. The bar inside the boxes represents the median value, the limits of the box the interquartile range, the whiskers the range of expected values and (·) are outlier values. **D-G**. Analysis of *SET*, *RUNX1*, *GATA2* and *MYC* expression in published high throughput data of adult *de novo* AML patients. mRNA expression values were extracted from data generated by the TCGA Research Network: http://cancergenome.nih.gov/. We analyzed the whole transcriptome of 182 AML samples using the Affymetrix HGU 133 Plus 2 microarray experiment provided by the TCGA Research Network: http://cancergenome.nih.gov/. R/Bioconductor [72] was used for the background correction and normalization of the samples using Robust Multichip Average (RMA) algorithm [73]. To filter out low expression probes, only those with log2-expression values above 5 in at least 30% of the samples were analyzed. In addition, the probesets 210231_x_at and 200630_x_at were removed from *SET* expression calculations due to their predicted unspecific partial binding to a transcribed region in chromosome 1. To calculate the expression values of each gene, the probesets that remained after these filtering steps were averaged. To arrange the samples by *SET* expression, this averaged value was used to set a percentile 70 thresholds, over which *SET* expression was considered high. Statistical analyses were performed with SPSS 17.0: *RUNX1*, *GATA2* and *MYC* expression levels were tested for correlation with *SET* expression using the Spearman's correlation test, and also compared between the high and low SET expression groups of patients by means of Student's t-test or Mann-Whitney's U test, depending on the distribution of each variable. Differences were considered significant when *p* < 0.05. The levels of mRNA expression in the SET high and SET low groups are represented for *SET* (D), *RUNX1* (E), *GATA2* (F), and *MYC* (G). The bar inside the boxes represents the median value, the limits of the box the interquartile range, the whiskers the range of expected values and (·) are outlier values. Correlation plots for *SET* expression vs. *RUNX1*, *GATA2* and *MYC* expression levels are also shown (right panels).

To evaluate the clinical relevance of our results, we analyzed the data from a series of patients with *de novo* AML that had been previously reported [8, 52]. We found a positive correlation between *SET* and *GATA2* expression levels in 167 patients (R= 0.256, *p*= 0.001; Figure 8A-8C), corroborating the association between these two genes in AML. We next investigated the expression of *SET*, *RUNX1*, *GATA2*, *SP1* and *MYC* in 182 samples of adult patients with *de novo* AML recently reported by the Cancer Genome Atlas [53]. There was a positive correlation between *SET* and either *RUNX1* (R=0.437, *p* < 0.001) or *GATA2* (R=0.339, *p* < 0.001), though the strongest significance was obtained when comparing *SET* and *MYC* expression (R=0.505, *p* < 0.001; (Figure 8D–8G). There was no significant correlation between *SET* and *SP1* levels (R= -0.089, *p*= 0.234; data not shown).

## DISCUSSION

Despite the importance of *SET* in both solid and hematologic tumors, little is known about the mechanisms involved in the transcriptional regulation of this oncogene. Here, we have studied the promoter region of *SET* in order to investigate the mechanisms that lead to *SET* overexpression in AML. We have characterized for the first time a minimal promoter region in the human *SET* gene, and we have demonstrated that MYC, SP1, RUNX1 and GATA2 form a multi-protein transcriptional complex that is involved in the transcriptional activation of this gene.

In this report, we have first confirmed that SET is an important regulator of proliferation and apoptosis in AML. Our experiments with shRNA and siRNAs showed the function of SET as an inducer of cell proliferation (by activating AKT and ERK pathways), and as an inhibitor of apoptosis in AML cells, through its binding and inhibition of PP2A. Although other *in vivo* model should be used to address the role of SET in leukemogenesis, these results are consistent with published data in chronic myeloid leukemia [5, 54] and other human cancer cells [5, 7, 8, 37, 39, 55], and strongly suggest that *SET* deregulation may play an important role in the pathogenesis of leukemia in AML patients.

We and others have previously reported that *SET* overexpression is associated with poor prognosis in AML and other malignancies [5, 7, 8, 37]. Despite these findings, which underscore the clinical relevance of *SET* expression, little is known about the causes of its overexpression in cancer. This prompted us to analyze the transcriptional regulation of *SET*. Here, we have defined the *SET* minimal promoter region (-318bp before the TSS), and found that this region is bound and activated by MYC and SP1, together with RUNX1 and GATA2. *In silico* analysis found no DNA motifs for RUNX1 and GATA2 in this region of SET promoter, and we hypothesized and confirmed that these TFs could form a complex with MYC and SP1. Furthermore, our results indicate that MYC is interacting with and recruiting SP1, RUNX1, and GATA2 on the chromatin; nevertheless, we cannot say whether they bind to the DNA directly or through MYC. Moreover, our results show that the depletion of any of these four TFs results in a significant reduction of *SET* expression. Previous separate studies have reported collaboration either between SP1 and MYC [46, 49, 50] or between SP1 and GATA2 [56, 57], or between RUNX1 and MYC [58]. Here, we have described the existence of a novel multi-protein complex in which RUNX1 and GATA2 interact with MYC and SP1, and activates *SET* transcription in AML cells. Additionally, our results prompt us to speculate about the presence of an intricate network of regulations among these TFs, in which MYC activates the expression of *SP1*, *RUNX1* and *GATA2*; RUNX1 and GATA2 increase the expression of *MYC*, and finally, SP1 increases the expression of *GATA2*. Likely, the expression of RUNX1 and GATA2 is affected by other proteins, or by a general lessening of cell proliferation, and not exclusively by MYC. Still, our data show that MYC is crucial for the recruitment of RUNX1, GATA2 and SP1 to the proximal promoter of *SET*. MYC is a well-known target of PP2A, since its protein stability is reduced by PP2A-dependent dephosphorylation of Serine 62 [59, 60]. Consequently, the impairment of PP2A activity by SET prevents MYC degradation [61]. Our study supports the importance of MYC as a potential target in AML, discovering its ability to reduce PP2A enzymatic activity through the regulation of *SET* transcription, and possibly CIP2A and SETBP1, defining a new layer of regulation that exists in the connection PP2A-MYC and SET-PP2A in cancer.

Finally, correlation analyses show that *SET* expression associates with *MYC*, *RUNX1* and *GATA2* expression in AML patients, corroborating our data and highlighting their clinical importance. However, the expression of *SP1* does not correlate with *SET* expression. The reason for this finding remains unclear. It has been reported that SP1 plays an important role in regulating cell proliferation by modulating the expression of several cell cycle regulatory proteins through specific sequences in G/C-rich promoter regions [62], and this is critical for transcriptional initiation of TATA-less promoters [63], such as the *SET* promoter. It can be hypothesized that SP1 acts as a housekeeping factor in AML cells, being constitutively present, and exerting different functions depending on the partners available to form multi-TF complexes. Supporting this hypothesis, the variability of *SP1* expression in the data series of The Cancer Genome Atlas that we have analyzed is quite low (CV= 22.2 %) compared to that of *MYC* (66.9%), *GATA2* (67.5%) and *RUNX1* (42.3%).

In summary, we have found that *SET* transcriptional regulation is controlled by at least four TFs. Interestingly, these are essential TFs that control different stages of hematopoiesis [42–45] and are frequently overexpressed in cancer [52, 62, 64–66]. Furthermore, our findings reveal a novel mechanism by which these TFs promote cancer. The simultaneous overexpression of MYC, SP1, RUNX1 and GATA2 would enhance the transcription of *SET*, which would trigger PP2A inactivation in AML, contributing to the leukemogenic phenotype. In this regard, treatments combining inhibitors for MYC, GATA2 and/or RUNX1 could be more effective and could minimize the development of resistance by targeting two or more proteins in the same complex. Further preclinical investigation of these possible combinatorial therapies in AML is still needed.

## MATERIALS AND METHODS

### Experimental animals

Rag2^-/-^γc^-/-^ mice were bred in house and treated according to the guidelines established by the University of Navarra Animal Experimentation Ethics Committee (No.:138-11). Stably transfected SET shRNA or control shRNA AML cells were harvested and re-suspended at 2 × 10^7^ cells per ml in sterile phosphate-buffered saline. Two groups (each group, n= 8) of 6 week-old female Rag2^-/-^γc^-/-^ mice were subcutaneously injected at the shoulder with 0.2 ml of the cell suspensions, one group for SET shRNA expressing cells and another for the control shRNA cells. Tumor growth was first measured 4 days after injection and then every day until the end of the experiment. Tumor volume (V) was monitored by measuring the length (L) and width (W) with calipers and calculated according to the formula (LxW^2^) x 0.5. After 10 days, tumor-bearing mice and controls were sacrificed and the tumors were excised and measured. Animals received a standard diet and water *ad libitum* at the Animal Core Facilities of the Center for Applied Medical Research (University of Navarra). All efforts were made to minimize the suffering of the animals.

### *In silico* analysis of the human promoter region of *SET*

For prediction of SET promoter and putative TF binding sites within the 2000 bp upstream the TSS of the SET transcript (NM_00320X), we used Promoter 2.0 [67], ElDorado (Genomatix Software, GmbH) PromoterScan (http://www-bimas.cit.nih.gov/molbio/proscan/), and NNPP (http://www.fruitfly.org/sequence/human-datasets.html) softwares. Then, the analysis was performed with MotifScanner program [68]. The promoter region was extracted from Ensembl database release 69 [69] and the position weight matrixes (PWM) of known transcription factor binding sites from the public versions of Jaspar [70] and Transfac [71] databases.

### Plasmid constructs and luciferase reporter assay

Promoter-luciferase reporter constructs were obtained by amplifying several regions of the SET promoter from BAC-RP11-216B9 (CHORI, BacPac Resources, Oakland, USA). The amplicons were digested and cloned into NheI–XhoI sites in the promoterless luciferase reporter vector (pGL3-Basic) (Promega, Madison, WI, USA). E-box mutants were obtained by triple ligation by using the primers listed in the Supplementary Table S1 and ligated in a NheI-ApaI digested -318-pGL3b vector. Single, double and triple mutants were tested. Luciferase activity was measured with Dual-Luciferase-Assay kit (Promega) 48h after transfection by electroporation of 3×10^6^ HL-60 or HEL cells with 5 μg of the promoter-luciferase construct and 1 μg of pRL-SV40. For the TF silencing experiments, the corresponding siRNAs for SP1, MYC, RUNX1, GATA2 or control were added to the electroporation mix. Firefly luciferase activity was normalized to *Renilla* luciferase signals to adjust the variability in transfection efficiencies. To study the effect of RUNX1 and GATA2 on the SET promoter, 100.000 HEK293t cells were transfected using the phosphate calcium method with 100 ng of pGL3-construct and 10 ng of pRL-SV40, together with 445 ng each of RUNX1 and GATA2 expression plasmids or 890 ng of empty vector.

### Chromatin immunoprecipitation

Chromatin from crosslinked HL-60 and HEL cells was sonicated, pre-cleared and incubated overnight with 3 mg of the corresponding antibody in RIPA buffer, and precipitated with protein G/A-Sepharose. For the re-ChIP assay, three fourth of the sample were incubated at 37°C for 30min in 10mM DTT. Supernatants were then incubated with the second antibody overnight, and protein G/A-Sepharose beads were added for two hour at 4°C the following day. The DNA-protein-antibody complexes were then washed three times with RIPA, three times with RIPA and NaCl, twice with Litium Buffer, and twice with 1X TE. Cross-linkage of the co-precipitated DNA–protein complexes was reversed and DNA was used as a template for quantitative Real Time PCR. Primers are listed in the Supplementary Table S1.

### Immunoprecipitation

Cell pellets were lysed in 3 ml of co-IP buffer (PBS containing 0.5% Triton X-100, 1mM EDTA, 100 μM sodium orthovanadate, 0.25 mM PMSF and complete protease inhibitor mixture, Roche). After centrifugation, protein samples were quantified by the BCA method. Overnight pre-clearing with non-specific IgG plus protein A/G beads was performed; then 1 mg protein was incubated with 5 μg of antibody crosslinked to protein A/G-Dynabeads following manufacturer's instructions (Invitrogen). The immunocomplexes were extensively washed with co-IP buffer and subsequently eluted with 50 μL of 0.1M Citrate pH 2.5 and boiled at 100°C in Laemmli buffer (2X) for Western blot analysis. IP control samples were incubated with irrelevant crosslinked IgG.

### Western blot analysis

For protein expression studies, 30 μg total protein lysates were separated in a 10-12% SDS-PAGE gradient gel and transferred onto a PVDF membrane. Overnight incubation at 4°C with primary antibodies was performed followed by the incubation with the appropriate secondary antibodies conjugated to horseradish peroxidase for 45′ at RT. The protein levels were detected by chemiluminescence (ECL kit, GE Healthcare, USA). The list of the primary antibodies used is described in the Supplementary Table S2.

### PP2A assay

PP2A activity was determined using the PP2A IP phosphatase assay kit (Upstate Biotechnology, 17-313). Briefly, protein lysates were prepared in TBS 1X, 1% Triton and phosphatase inhibitors tablets (Roche). Fifty micrograms of protein were immunoprecipitated with 2 μg of PP2A antibody (1D6; Upstate Biotechnology)and 25 μL of protein-A-agarose beads for 2 hours at 4°C. After extensive washing with TBS first and with Ser/Thr assay buffer last, the beads were then used in the phosphatase reaction for measuring dephosphorylation of the phosphopeptide (K-R-pT-I-R-R) with malachite green phosphate detection solution, following the manufacturer's instructions. The level of free phosphate is then normalized to total amount of immunoprecipitated PP2Ac, assessed by densitometry analysis of the western blots.

### Statistical analysis

Statistical significance of differences between means was estimated using Student's *t*-test, Mann-Whitney U test, Oneway or Twoway-ANOVA and Bonferroni test, as appropriate. To evaluate the correlation analysis between SET and TFs in AML patients, Spearman's rank correlation coefficient was used, because the values did not fit the normal distribution. For contingency studies on the distribution of frequencies of high or low expression of SET and GATA2 in AML patients, the Chi square test was applied. Statistical analysis was performed using the SPSS 17.0 statistical package (Chicago, IL, USA) and GraphPad software (Prism, USA). A *P*-value of 0.05 was considered to be significant (*), 0.01 or 0.001 very significant (** or ***).

## SUPPLEMENTARY MATERIALS FIGURES







## References

[1] Ravandi F, Cortes J, Faderl S, O’Brien S, Garcia-Manero G, Verstovsek S, Santos FP, Shan J, Brandt M, de Lima M, Pierce S, Kantarjian H (2010). Characteristics and outcome of patients with acute myeloid leukemia refractory to 1 cycle of high-dose cytarabine-based induction chemotherapy. Blood.

[2] Dores GM, Devesa SS, Curtis RE, Linet MS, Morton LM (2012). Acute leukemia incidence and patient survival among children and adults in the United States, 2001-2007. Blood.

[3] Döhner H, Weisdorf DJ, Bloomfield CD (2015). Acute Myeloid Leukemia. N Engl J Med.

[4] Perrotti D, Neviani P (2013). Protein phosphatase 2A: a target for anticancer therapy. Lancet Oncol.

[5] Neviani P, Santhanam R, Trotta R, Notari M, Blaser BW, Liu S, Mao H, Chang JS, Galietta A, Uttam A, Roy DC, Valtieri M, Bruner-Klisovic R (2005). The tumor suppressor PP2A is functionally inactivated in blast crisis CML through the inhibitory activity of the BCR/ABL-regulated SET protein. Cancer Cell.

[6] Cristobal I, Garcia-Orti L, Cirauqui C, Alonso MM, Calasanz MJ, Odero MD (2011). PP2A impaired activity is a common event in acute myeloid leukemia and its activation by forskolin has a potent anti-leukemic effect. Leukemia.

[7] Christensen DJ, Chen Y, Oddo J, Matta KM, Neil J, Davis ED, Volkheimer AD, Lanasa MC, Friedman DR, Goodman BK, Gockerman JP, Diehl LF, de Castro CM (2011). SET oncoprotein overexpression in B-cell chronic lymphocytic leukemia and non-Hodgkin lymphoma: a predictor of aggressive disease and a new treatment target. Blood.

[8] Cristobal I, Garcia-Orti L, Cirauqui C, Cortes-Lavaud X, Garcia-Sanchez MA, Calasanz MJ, Odero MD (2012). Overexpression of SET is a recurrent event associated with poor outcome and contributes to protein phosphatase 2A inhibition in acute myeloid leukemia. Haematologica.

[9] Canela N, Rodriguez-Vilarrupla A, Estanyol JM, Diaz C, Pujol MJ, Agell N, Bachs O (2003). The SET protein regulates G2/M transition by modulating cyclin B-cyclin-dependent kinase 1 activity. J Biol Chem.

[10] JP ten Klooster, Leeuwen I, Scheres N, Anthony EC, Hordijk PL (2007). Rac1-induced cell migration requires membrane recruitment of the nuclear oncogene SET. EMBO J.

[11] Fan Z, Beresford PJ, Oh DY, Zhang D, Lieberman J (2003). Tumor suppressor NM23-H1 is a granzyme A-activated DNase during CTL-mediated apoptosis, and the nucleosome assembly protein SET is its inhibitor. Cell.

[12] Madeira A, Pommet JM, Prochiantz A, Allinquant B (2005). SET protein (TAF1beta, I2PP2A) is involved in neuronal apoptosis induced by an amyloid precursor protein cytoplasmic subdomain. FASEB J.

[13] Samanta AK, Chakraborty SN, Wang Y, Kantarjian H, Sun X, Hood J, Perrotti D, Arlinghaus RB (2009). Jak2 inhibition deactivates Lyn kinase through the SET-PP2A-SHP1 pathway, causing apoptosis in drug-resistant cells from chronic myelogenous leukemia patients. Oncogene.

[14] Kim DW, Kim KB, Kim JY, Lee KS, Seo SB (2010). Negative regulation of neuronal cell differentiation by INHAT subunit SET/TAF-Ibeta. Biochem Biophys Res Commun.

[15] Kalousi A, Hoffbeck AS, Selemenakis PN, Pinder J, Savage KI, Khanna KK, Brino L, Dellaire G, Gorgoulis VG, Soutoglou E (2015). The nuclear oncogene SET controls DNA repair by KAP1 and HP1 retention to chromatin. Cell Rep.

[16] Nagata K, Kawase H, Handa H, Yano K, Yamasaki M, Ishimi Y, Okuda A, Kikuchi A, Matsumoto K (1995). Replication factor encoded by a putative oncogene, set, associated with myeloid leukemogenesis. Proc Natl Acad Sci U S A.

[17] Seo SB, McNamara P, Heo S, Turner A, Lane WS, Chakravarti D (2001). Regulation of histone acetylation and transcription by INHAT, a human cellular complex containing the set oncoprotein. Cell.

[18] Cervoni N, Detich N, Seo SB, Chakravarti D, Szyf M (2002). The oncoprotein Set/TAF-1beta, an inhibitor of histone acetyltransferase, inhibits active demethylation of DNA, integrating DNA methylation and transcriptional silencing. J Biol Chem.

[19] Macfarlan T, Parker JB, Nagata K, Chakravarti D (2006). Thanatos-associated protein 7 associates with template activating factor-Ibeta and inhibits histone acetylation to repress transcription. Mol Endocrinol.

[20] Kim JY, Lee KS, Seol JE, Yu K, Chakravarti D, Seo SB (2012). Inhibition of p53 acetylation by INHAT subunit SET/TAF-Ibeta represses p53 activity. Nucleic Acids Res.

[21] Kato K, Okuwaki M, Nagata K (2011). Role of Template Activating Factor-I as a chaperone in linker histone dynamics. J Cell Sci.

[22] Leung JW, Leitch A, Wood JL, Shaw-Smith C, Metcalfe K, Bicknell LS, Jackson AP, Chen J (2011). SET nuclear oncogene associates with microcephalin/MCPH1 and regulates chromosome condensation. J Biol Chem.

[23] Adachi Y, Pavlakis GN, Copeland TD (1994). Identification and characterization of SET, a nuclear phosphoprotein encoded by the translocation break point in acute undifferentiated leukemia. J Biol Chem.

[24] Li M, Makkinje A, Damuni Z (1996). The myeloid leukemia-associated protein SET is a potent inhibitor of protein phosphatase 2A. J Biol Chem.

[25] Arnaud L, Chen S, Liu F, Li B, Khatoon S, Grundke-Iqbal I, Iqbal K (2011). Mechanism of inhibition of PP2A activity and abnormal hyperphosphorylation of tau by I2(PP2A)/SET. FEBS Lett.

[26] Pippa R, Dominguez A, Christensen DJ, Moreno-Miralles I, Blanco-Prieto MJ, Vitek MP, Odero MD (2014). Effect of FTY720 on the SET-PP2A complex in acute myeloid leukemia; SET binding drugs have antagonistic activity. Leukemia.

[27] Arif M, Wei J, Zhang Q, Liu F, Basurto-Islas G, Grundke-Iqbal I, Iqbal K (2014). Cytoplasmic retention of protein phosphatase 2A inhibitor 2 (I2PP2A) induces Alzheimer-like abnormal hyperphosphorylation of Tau. J Biol Chem.

[28] Qu D, Zhang Y, Ma J, Guo K, Li R, Yin Y, Cao X, Park DS (2007). The nuclear localization of SET mediated by impalpha3/impbeta attenuates its cytosolic toxicity in neurons. J Neurochem.

[29] Lam BD, Anthony EC, Hordijk PL (2013). Cytoplasmic targeting of the proto-oncogene SET promotes cell spreading and migration. FEBS Lett.

[30] Walker CJ, Oaks JJ, Santhanam R, Neviani P, Harb JG, Ferenchak G, Ellis JJ, Landesman Y, Eisfeld AK, Gabrail NY, Smith CL, Caligiuri MA, Hokland P (2013). Preclinical and clinical efficacy of XPO1/CRM1 inhibition by the karyopherin inhibitor KPT-330 in Ph+ leukemias. Blood.

[31] Vasudevan NT, Mohan ML, Gupta MK, Hussain AK, Naga Prasad SV (2011). Inhibition of protein phosphatase 2A activity by PI3Kgamma regulates beta-adrenergic receptor function. Mol Cell.

[32] Yu G, Yan T, Feng Y, Liu X, Xia Y, Luo H, Wang JZ, Wang X (2013). Ser9 phosphorylation causes cytoplasmic detention of I2PP2A/SET in Alzheimer disease. Neurobiol Aging.

[33] Neviani P, Santhanam R, Oaks JJ, Eiring AM, Notari M, Blaser BW, Liu S, Trotta R, Muthusamy N, Gambacorti-Passerini C, Druker BJ, Cortes J, Marcucci G (2007). FTY720, a new alternative for treating blast crisis chronic myelogenous leukemia and Philadelphia chromosome-positive acute lymphocytic leukemia. J Clin Invest.

[34] Christensen DJ, Ohkubo N, Oddo J, Van Kanegan MJ, Neil J, Li F, Colton CA, Vitek MP (2011). Apolipoprotein E and peptide mimetics modulate inflammation by binding the SET protein and activating protein phosphatase 2A. J Immunol.

[35] Oaks JJ, Santhanam R, Walker CJ, Roof S, Harb JG, Ferenchak G, Eisfeld AK, Van Brocklyn JR, Briesewitz R, Saddoughi SA, Nagata K, Bittman R, Caligiuri MA (2013). Antagonistic activities of the immunomodulator and PP2A-activating drug FTY720 (Fingolimod, Gilenya) in Jak2-driven hematologic malignancies. Blood.

[36] Saddoughi SA, Gencer S, Peterson YK, Ward KE, Mukhopadhyay A, Oaks J, Bielawski J, Szulc ZM, Thomas RJ, Selvam SP, Senkal CE, Garrett-Mayer E, De Palma RM (2013). Sphingosine analogue drug FTY720 targets I2PP2A/SET and mediates lung tumour suppression via activation of PP2A-RIPK1-dependent necroptosis. EMBO Mol Med.

[37] Farrell AS, Allen-Petersen B, Daniel CJ, Wang X, Wang Z, Rodriguez S, Impey S, Oddo J, Vitek MP, Lopez C, Christensen DJ, Sheppard B, Sears RC (2014). Targeting Inhibitors of the Tumor Suppressor PP2A for the Treatment of Pancreatic Cancer. Mol Cancer Res.

[38] Janghorban M, Farrell AS, Allen-Petersen BL, Pelz C, Daniel CJ, Oddo J, Langer EM, Christensen DJ, Sears RC (2014). Targeting c-MYC by antagonizing PP2A inhibitors in breast cancer. Proc Natl Acad Sci U S A.

[39] Cristobal I, Rincon R, Manso R, Carames C, Zazo S, Madoz-Gurpide J, Rojo F, Garcia-Foncillas J (2015). Deregulation of the PP2A inhibitor SET shows promising therapeutic implications and determines poor clinical outcome in patients with metastatic colorectal cancer. Clin Cancer Res.

[40] Junttila MR, Puustinen P, Niemela M, Ahola R, Arnold H, Bottzauw T, Ala-aho R, Nielsen C, Ivaska J, Taya Y, Lu SL, Lin S, Chan EK (2007). CIP2A inhibits PP2A in human malignancies. Cell.

[41] Cristobal I, Blanco FJ, Garcia-Orti L, Marcotegui N, Vicente C, Rifon J, Novo FJ, Bandres E, Calasanz MJ, Bernabeu C, Odero MD (2010). SETBP1 overexpression is a novel leukemogenic mechanism that predicts adverse outcome in elderly patients with acute myeloid leukemia. Blood.

[42] Delgado MD, Leon J (2010). Myc roles in hematopoiesis and leukemia. GenesCancer.

[43] Vicente C, Conchillo A, Garcia-Sanchez MA, Odero MD (2012). The role of the GATA2 transcription factor in normal and malignant hematopoiesis. Crit Rev Oncol Hematol.

[44] Ichikawa M, Yoshimi A, Nakagawa M, Nishimoto N, Watanabe-Okochi N, Kurokawa M (2013). A role for RUNX1 in hematopoiesis and myeloid leukemia. Int J Hematol.

[45] Gilmour J, Assi SA, Jaegle U, Kulu D, van de Werken H, Clarke D, Westhead DR, Philipsen S, Bonifer C (2014). A crucial role for the ubiquitously expressed transcription factor Sp1 at early stages of hematopoietic specification. Development.

[46] Gartel AL, Ye X, Goufman E, Shianov P, Hay N, Najmabadi F, Tyner AL (2001). Myc represses the p21(WAF1/CIP1) promoter and interacts with Sp1/Sp3. Proc Natl Acad Sci U S A.

[47] Koshiji M, To KK, Hammer S, Kumamoto K, Harris AL, Modrich P, Huang LE (2005). HIF-1alpha induces genetic instability by transcriptionally downregulating MutSalpha expression. Mol Cell.

[48] Maiques-Diaz A, Chou FS, Wunderlich M, Gomez-Lopez G, Jacinto FV, Rodriguez-Perales S, Larrayoz MJ, Calasanz MJ, Mulloy JC, Cigudosa JC, Alvarez S (2012). Chromatin modifications induced by the AML1-ETO fusion protein reversibly silence its genomic targets through AML1 and Sp1 binding motifs. Leukemia.

[49] Gopisetty G, Xu J, Sampath D, Colman H, Puduvalli VK (2013). Epigenetic regulation of CD133/PROM1 expression in glioma stem cells by Sp1/myc and promoter methylation. Oncogene.

[50] You X, Liu F, Zhang T, Lv N, Liu Q, Shan C, Du Y, Kong G, Wang T, Ye L, Zhang X (2014). Hepatitis B virus X protein upregulates Lin28A/Lin28B through Sp-1/c-Myc to enhance the proliferation of hepatoma cells. Oncogene.

[51] Wilson NK, Foster SD, Wang X, Knezevic K, Schutte J, Kaimakis P, Chilarska PM, Kinston S, Ouwehand WH, Dzierzak E, Pimanda JE, de Bruijn MF, Gottgens B (2010). Combinatorial transcriptional control in blood stem/progenitor cells: genome-wide analysis of ten major transcriptional regulators. Cell Stem Cell.

[52] Vicente C, Vazquez I, Conchillo A, Garcia-Sanchez MA, Marcotegui N, Fuster O, Gonzalez M, Calasanz MJ, Lahortiga I, Odero MD (2012). Overexpression of GATA2 predicts an adverse prognosis for patients with acute myeloid leukemia and it is associated with distinct molecular abnormalities. Leukemia.

[53] Cancer Genome Atlas Research N. Genomic and epigenomic landscapes of adult de novo acute myeloid leukemia (2013). N Engl J Med.

[54] Agarwal A, MacKenzie RJ, Pippa R, Eide CA, Oddo J, Tyner JW, Sears R, Vitek MP, Odero MD, Christensen DJ, Druker BJ (2014). Antagonism of SET Using OP449 Enhances the Efficacy of Tyrosine Kinase Inhibitors and Overcomes Drug Resistance in Myeloid Leukemia. Clin Cancer Res.

[55] Carlson SG, Eng E, Kim EG, Perlman EJ, Copeland TD, Ballermann BJ (1998). Expression of SET, an inhibitor of protein phosphatase 2A, in renal development and Wilms’ tumor. J Am Soc Nephrol.

[56] Merika M, Orkin SH (1995). Functional synergy and physical interactions of the erythroid transcription factor GATA-1 with the Kruppel family proteins Sp1 and EKLF. Mol Cell Biol.

[57] Maeda K, Nishiyama C, Ogawa H, Okumura K (2010). GATA2 and Sp1 positively regulate the c-kit promoter in mast cells. J Immunol.

[58] Agrawal P, Yu K, Salomon AR, Sedivy JM (2010). Proteomic profiling of Myc-associated proteins. Cell Cycle.

[59] Yeh E, Cunningham M, Arnold H, Chasse D, Monteith T, Ivaldi G, Hahn WC, Stukenberg PT, Shenolikar S, Uchida T, Counter CM, Nevins JR, Means AR, Sears R (2004). A signalling pathway controlling c-Myc degradation that impacts oncogenic transformation of human cells. Nat Cell Biol.

[60] Arnold HK, Sears RC (2006). Protein phosphatase 2A regulatory subunit B56alpha associates with c-myc and negatively regulates c-myc accumulation. Mol Cell Biol.

[61] Mukhopadhyay A, Saddoughi SA, Song P, Sultan I, Ponnusamy S, Senkal CE, Snook CF, Arnold HK, Sears RC, Hannun YA, Ogretmen B (2009). Direct interaction between the inhibitor 2 and ceramide via sphingolipid-protein binding is involved in the regulation of protein phosphatase 2A activity and signaling. FASEB J.

[62] Li L, Davie JR (2010). The role of Sp1 and Sp3 in normal and cancer cell biology. Ann Anat.

[63] Wierstra I (2008). Sp1: Emerging roles - Beyond constitutive activation of TATA-less housekeeping genes. Biochem Biophys Res Commun.

[64] Junttila MR, Westermarck J (2008). Mechanisms of MYC stabilization in human malignancies. Cell Cycle.

[65] Liu X, Zhang Q, Zhang DE, Zhou C, Xing H, Tian Z, Rao Q, Wang M, Wang J (2009). Overexpression of an isoform of AML1 in acute leukemia and its potential role in leukemogenesis. Leukemia.

[66] Wang W, Schwemmers S, Hexner EO, Pahl HL (2010). AML1 is overexpressed in patients with myeloproliferative neoplasms and mediates JAK2V617F-independent overexpression of NF-E2. Blood.

[67] Knudsen S (1999). Promoter2.0: for the recognition of PolII promoter sequences. Bioinformatics.

[68] Coessens B, Thijs G, Aerts S, Marchal K, De Smet F, Engelen K, Glenisson P, Moreau Y, Mathys J, De Moor B (2003). INCLUSive: A web portal and service registry for microarray and regulatory sequence analysis. Nucleic Acids Res.

[69] Flicek P, Amode MR, Barrell D, Beal K, Billis K, Brent S, Carvalho-Silva D, Clapham P, Coates G, Fitzgerald S, Gil L, Giron CG, Gordon L (2014). Ensembl.

[70] Mathelier A, Zhao X, Zhang AW, Parcy F, Worsley-Hunt R, Arenillas DJ, Buchman S, Chen CY, Chou A, Ienasescu H, Lim J, Shyr C, Tan G (2014). an extensively expanded and updated open-access database of transcription factor binding profiles. JASPAR.

[71] Matys V, Kel-Margoulis OV, Fricke E, Liebich I, Land S, Barre-Dirrie A, Reuter I, Chekmenev D, Krull M, Hornischer K, Voss N, Stegmaier P, Lewicki-Potapov B (2006). TRANSFAC and its module TRANSCompel: transcriptional gene regulation in eukaryotes. Nucleic Acids Res.

[72] Gentleman RC, Carey VJ, Bates DM, Bolstad B, Dettling M, Dudoit S, Ellis B, Gautier L, Ge Y, Gentry J, Hornik K, Hothorn T, Huber W (2004). Bioconductor: open software development for computational biology and bioinformatics. Genome Biol.

[73] Irizarry RA, Bolstad BM, Collin F, Cope LM, Hobbs B, Speed TP (2003). Summaries of Affymetrix GeneChip probe level data. Nucleic Acids Res.

