# Supplementary material for: MYC-dependent recruitment of RUNX1 and GATA2 on the SET oncogene promoter enhances PP2A inactivation in acute myeloid leukemia
Source: Oncotarget. 2016 Jun 6;8(33):53989–4003. doi: 10.18632/oncotarget.9840 (PMC5589557; doi:10.18632/oncotarget.9840)
Supplement: Supplementary file 1 [file oncotarget-08-53989-s001.pdf]

MYC-dependent recruitment of RUNX1 and GATA2 on the SET oncogene promoter enhances PP2A inactivation in acute myeloid leukemia

SUPPLEMENTARY MATERIALS AND METHODS

Cell lines culture

HL-60, HEL, and A549 cells were maintained in RPMI-1640 (Invitrogen) with 10% fetal bovine serum (Life Technologies). NIH3T3, HEK293t and HeLa cells were grown in DMEM supplemented with 10% fetal calf serum (Life Technologies). Cell lines were grown at 37°C in a 5% CO_2 atmosphere. Media were supplemented with penicillin G (100 U/mL), and streptomycin (0.1 mg/mL).

shRNA stable cells

HEK293t cells were transduced with scramble shRNA (sc-108080) and a specific SET shRNA with the following hairpin sequence: 5′-GATCCAAATAAAGTT CTCTCCAAATTCAAGAGATTTGGAGAGAACTTTA TTTTTTTT-3′, (sc-43856, both from Santa Cruz, USA). The virus-loaded supernatant obtained was used to infect AML cells. After 72h, fresh media were added to the cells and they were maintained for one week in presence of puromycin (Sigma) until the collection of the samples.

siRNA transient transfections

To knock down SET expression, three different siRNAs for SET inhibition were tested. The siRNA previously described (37) with the following sequence 5′-AAACGUUCGAGUCAAACGCAG -3′, was the most effective at the concentration of 400 pmol and was used for the experiments. For the depletion of RUNX1, GATA2, SP1 and MYC in AML cells, we used the following siRNAs: ON-TARGETplus SMARTpool siRNA L-003926-00-0005 for RUNX1 (GE Healthcare Dharmacon), GATA2 stealth 1299001 for GATA2 (Life Technologies), SP1 Santa Cruz siRNA sc-29487 (siSP1 #2) or custom designed SP1 (siSP1 #1) and MYC siRNAs with the following sequence: 5′-AAGGAGUUGGUGGCAAUAA-3′ and 5′-UCCUGAGACAGAUCAGCAACAACCG- 3′ respectively. siRNA transfection was performed by electroporation with the Gene-Pulser-Xcell Electroporation System (Bio-Rad, Benicia, CA, USA) using 300 V and a capacitance of 1000 mF for HL-60; 395V and 950mF for HEL. Calcium Phosphate transfection method was used for HEK293t cells.

Cell proliferation

Cell proliferation was determined by MTS assay (Promega, USA) following the manufacturer's instructions. The cell growth curve was generated accordingly, harvesting the cells at 24h intervals during 72 or 96h.

Colony formation assay

Cells were plated in triplicate in six-well plates with agarose (Pronadisa) (top layer 0.3%; bottom layer 0.6%) and incubated until colonies were visible (10 days). The colonies were stained by adding 500 µL of 5 mg/mL MTT reagent (3-(4,5-Dimethylthiazol-2-yl)-2,5-diphenyltetrazolium bromide, Sigma) during 4h at 37°C. Colonies were fixed by overnight incubation with DMSO at 37°C and their number was assessed using Image J software (NIH, USA).

Assessment of apoptosis by flow cytometry

Flow cytometry, after staining with Annexin V and Propidium Iodide (PI), was used to determine the level of early and late-stage apoptosis following the manufacturer's protocol (BD Biosciences). Briefly, PBS washed cells were suspended in a total volume of 100 µl binding buffer (1X) and incubated with 5 µl Annexin V and 5 µl PI during 15 min in the dark at room temperature. After this incubation, the cells were suspended to a total volume of 500 µl and analyzed with a flow cytometer (Becton Dickinson FACScalibur). Data analysis was performed with CellQuest software. For the graphical representation of apoptosis data, the sum of the percentages of early and late cell death (Annexin V positive cells) was used.

RNA retrotranscription and quantitative real time PCR (qRT-PCR)

RNA was isolated with RNeasy-Mini-Kit (Qiagen, USA) and 2 µg were used for cDNA synthesis with the High Capacity cDNA Reverse Transcription Kit (Applied Biosystems, USA). qRT–PCR was performed with 20 ng of cDNA in the ABI-Prism-7500 (Applied Biosystems, USA) using specific primers for SET and HPRT (housekeeping), as listed in the Supplementary Table S1.

REFERENCES

1. 1. Kent WJ, Sugnet CW, Furey TS, Roskin KM, Pringle TH, Zahler AM, Haussler D. The human genome browser at UCSC. Genome Res. 2002;12:996-1006.

SUPPLEMENTARY FIGURES AND TABLES

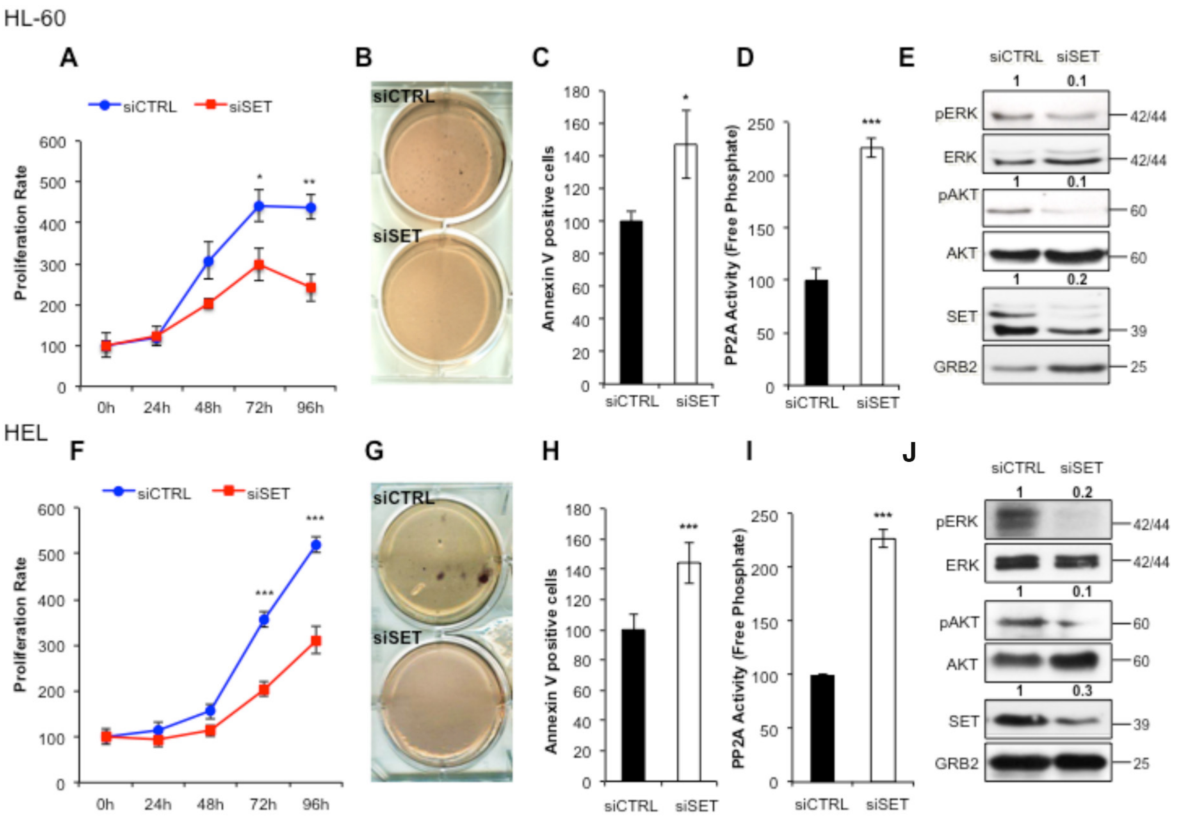

Supplementary Figure S1: Transient depletion of SET provokes a reduction of cell proliferation and re-establishes PP2A activity in AML cell lines. A, F. Cell proliferation rate and **B, G.** soft-agar growth in cells transfected with SET siRNA (siSET) compared with siRNA control (siCTRL). **C, H.** Percentage of Annexin V positive cells and **D, I.** PP2A activity 72h after transfection. **E, J.** Western blot showing the levels of SET and phosphorylation state of PP2A targets ERK and AKT in cells transfected with SET siRNA versus control. HL-60 **A-E.** and HEL **F-J.** cell lines were used. Numbers indicate the protein quantification relative to GRB2 and assessed using Image J software (NIH, USA). Values are the mean ± SD of three independent experiments. Statistically significant differences are indicated: *P < 0.05, **P < 0.01, ***P < 0.001, Student's t-test analysis.

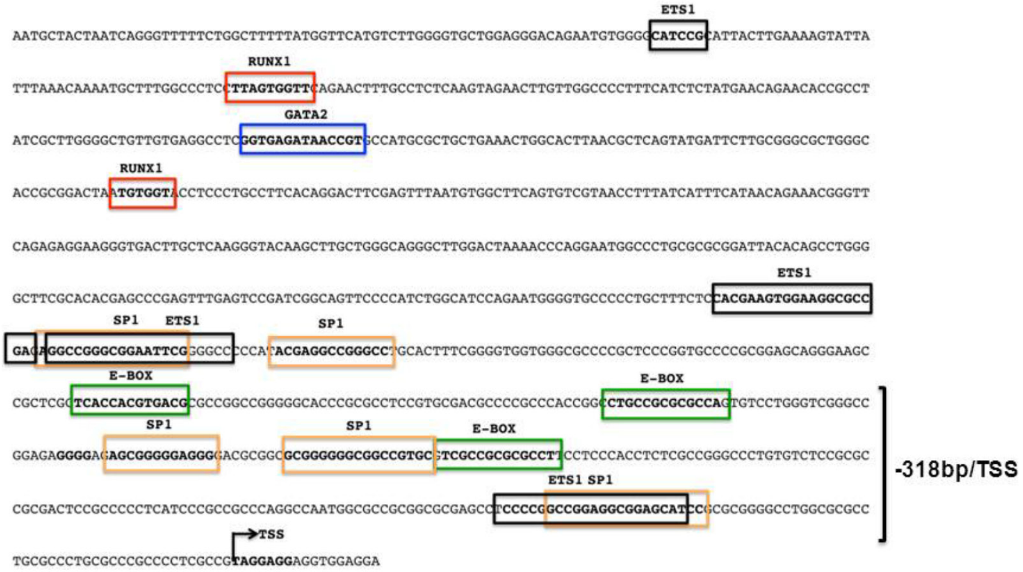

Supplementary Figure S2: Transcription factors consensus site analysis in the SET promoter. Detailed representation of the predicted TFBS within the sequence of the *SET* promoter region.

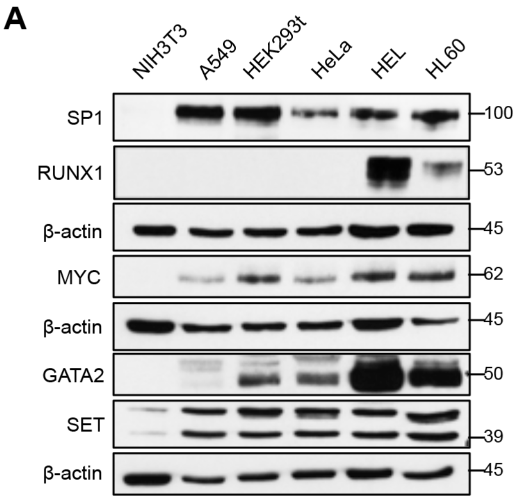

Supplementary Figure S3: Western blot showing the endogenous level of SET, RUNX1, GATA2, SP1, and MYC in NIH3T3, A549, HEK293t, HeLa and AML cell lines HEL and HL-60. *β*-Actin was used as loading control.

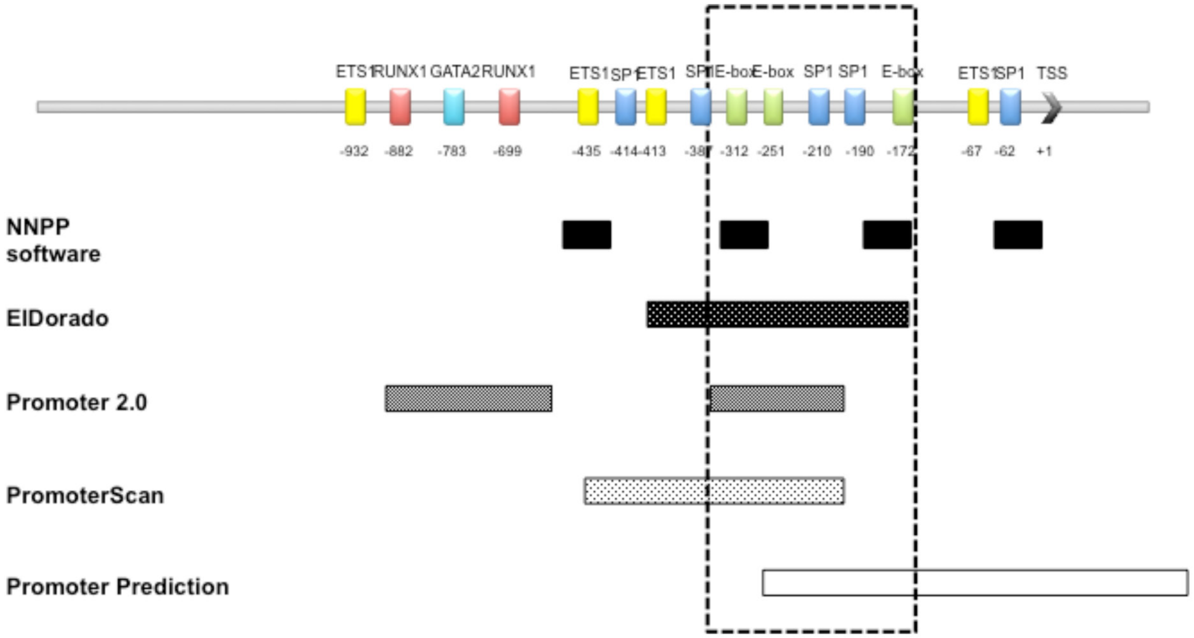

Supplementary Figure S4: *In silico* analysis of the SET promoter. Schematic representation of the genomic sequence analysis obtained using the following bioinformatics algorithms: NNPP, ElDorado (which estimate transcription factors binding sites, TFBS), PromoterScan, Promoter 2.0 (which analyze the possible RNApol II binding) and Genome Browser (Kent et al., 2002). A discontinuous line encompasses the region most often predicted as promoter by the different approaches. Predicted transcription factors binding sites (TFBS) are indicated with colors.

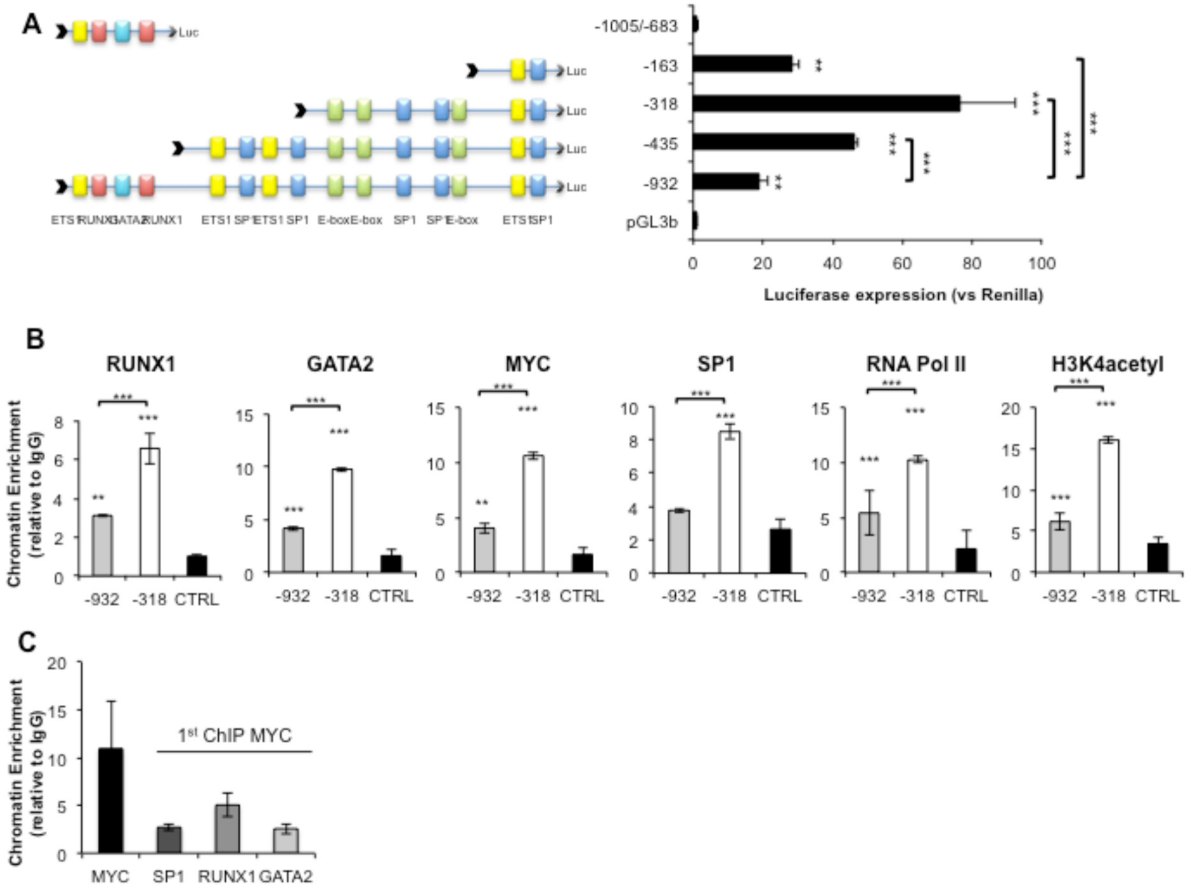

Supplementary Figure S5: RUNX1, GATA2, SP1 and MYC co-localize on SET minimal functional promoter region (-318bp/TSS). A. Luciferase assays with the *SET* promoter constructs in HEL cells. Relative Firefly/Renilla luciferase activities considering the empty pGL3basic as 1 are represented. Values are the mean ± SD of three independent experiments. Asterisks indicate the significance between the different constructs. *P < 0.05, **P < 0.01, ***P < 0.001, Two-way ANOVA and Bonferroni post-hoc tests were used. **B.** Chromatin Immunoprecipitation assay assessing the fold enrichment in the binding of the analyzed TFs to the (-933/-587bp) and (-318/-144bp) regions compared with a distal genomic sequence on the same chromosome 9 used as a negative control (CTRL). qRT–PCR results were calculated using the 2-ΔΔCt method and they are presented as the fold enrichment of chromatin DNA precipitated by the specific antibody versus chromatin DNA precipitated by normal IgG. Values are the mean ± SD of two independent experiments. Statistical significant differences are indicated: *P < 0.05, **P < 0.01, ***P < 0.001. **C.** ChIP-re-ChIP assay performed in the HEL. Technical procedures were carried out as described in Materials and Methods. MYC antibody was used for the first immunoprecipitation, and SP1, RUNX1, and GATA2 antibodies were used for the second immunoprecipitation. Re-ChIP assay Values are the mean of two independent experiments.

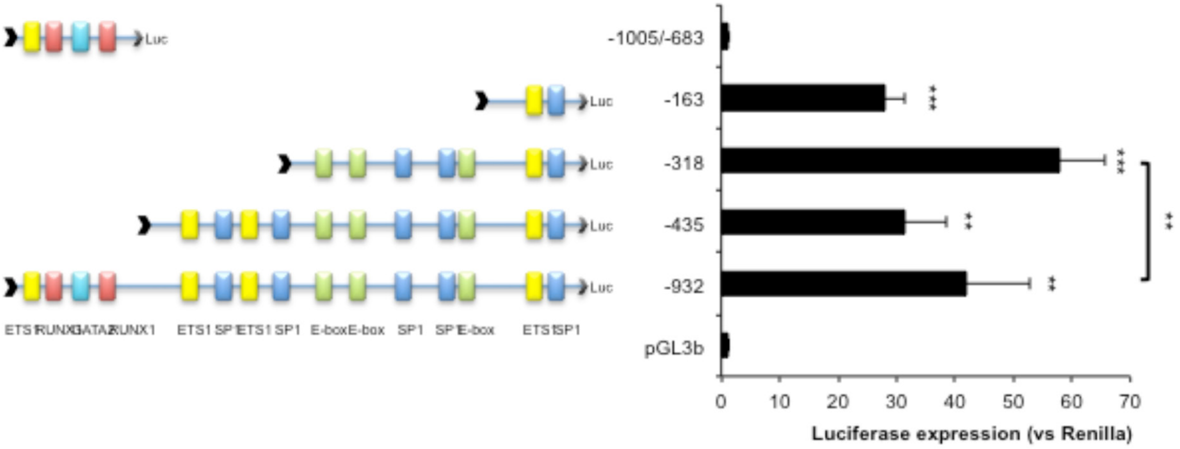

Supplementary Figure S6: Minimal functional SET promoter region in HEK293t cells. Luciferase assays with the *SET* promoter constructs in HEK293t cells. Relative Firefly/Renilla luciferase activities considering the empty pGL3basic as 1 are represented. Values are the mean ± SD of three independent experiments. Asterisks indicate the significance between the different constructs. Statistically analysis was performed with Kruskal-Wallis test followed by Mann-Whitney's U test corrected for multiple comparisons by Holm-Bonferroni. Statistical significant differences are indicated: *P < 0.05, **P < 0.01, ***P < 0.001.

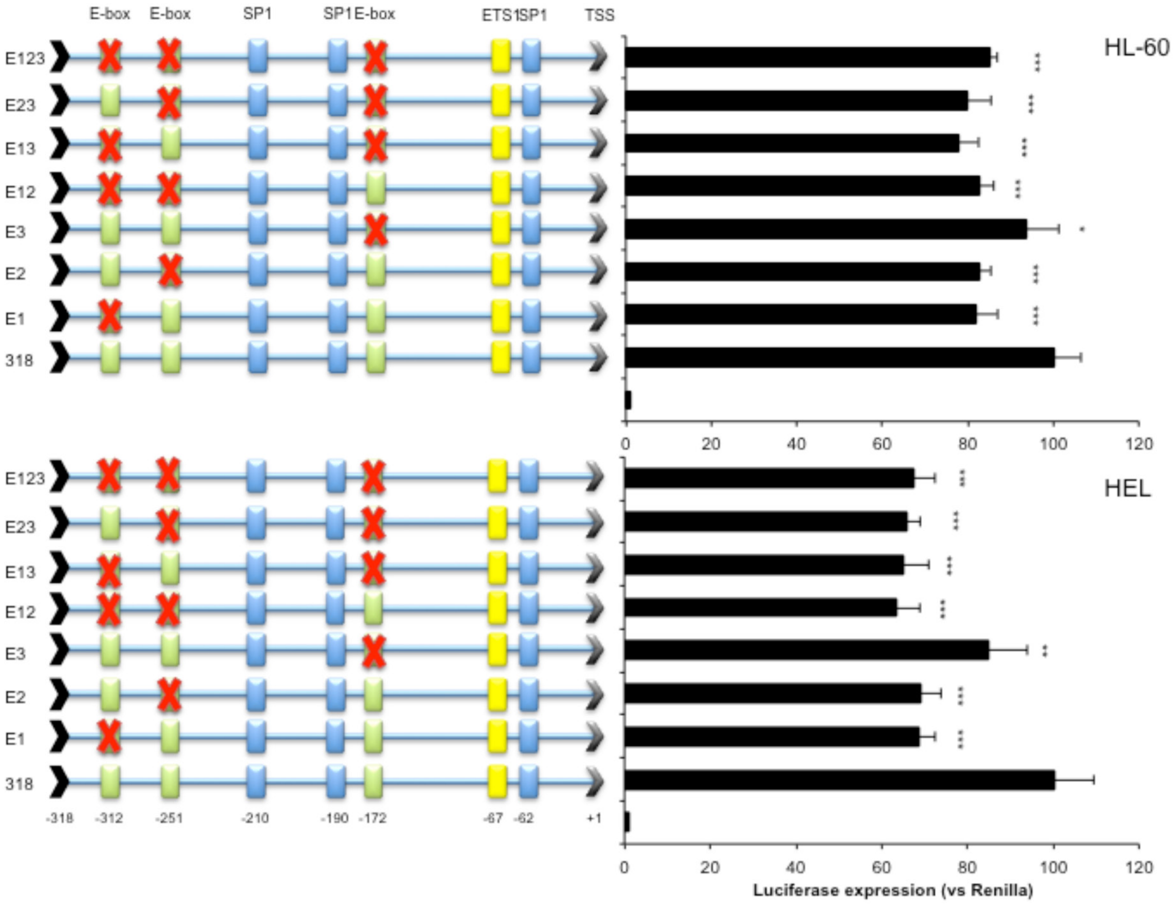

Supplementary Figure S7: E-box binding region for SET promoter activity in AML. Luciferase assays of the 318-bp region with the predicted E-box sites mutated in the HL-60 (upper panel) and HEL (lower panel) cell lines. Results represent relative Firefly/Renilla luciferase activities considering the WT 318 bp region as 100%. Data are the means ± SD of three independent experiments. *P < 0.05, **P < 0.01, ***P < 0.001, Students t-test analysis.

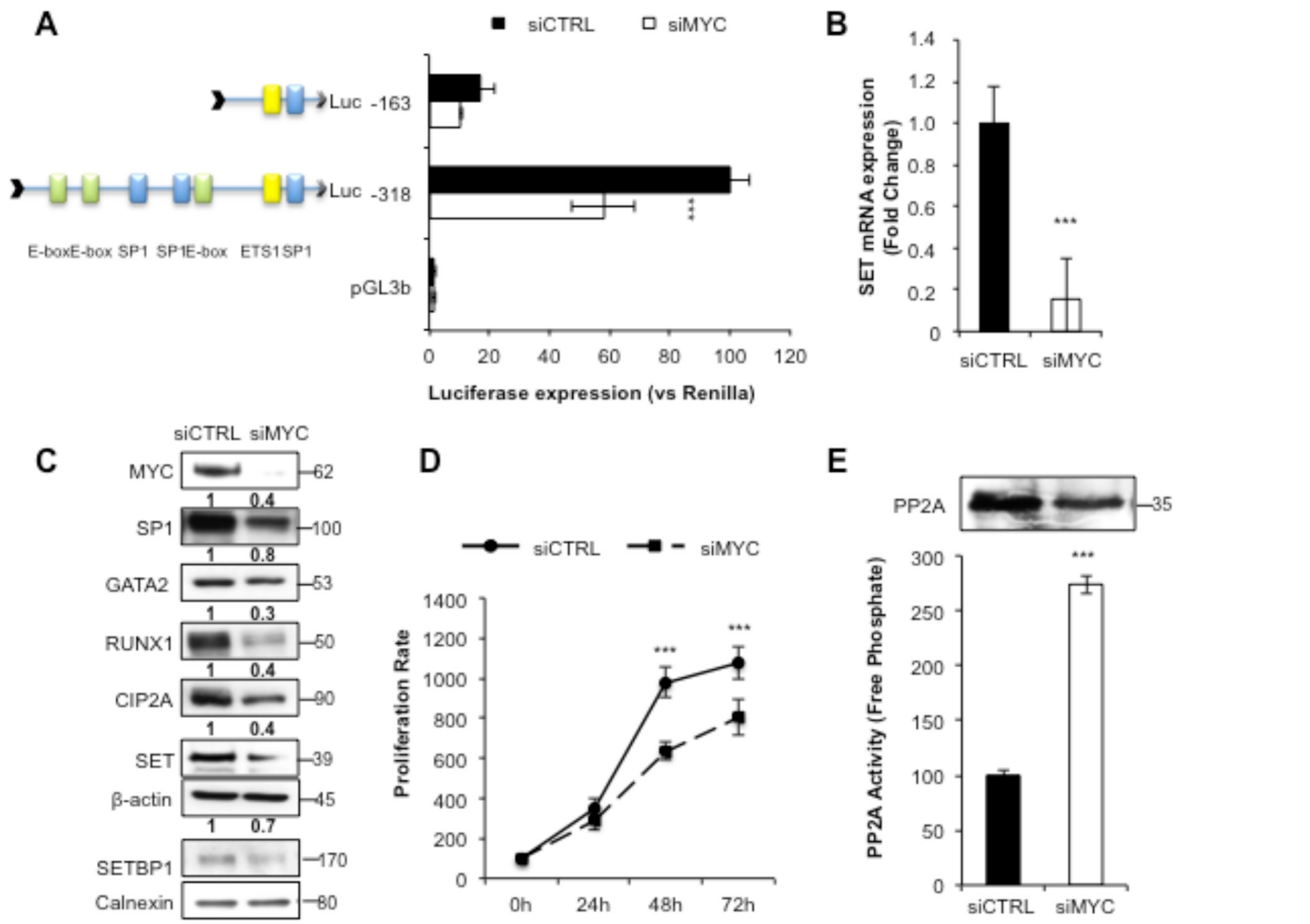

Supplementary Figure S8: MYC depletion significantly reduces SET transcription and the re-activates PP2A function in AML. Luciferase assay in HEL cells transfected with siRNA of MYC (siMYC). **B.** *SET* mRNA expression assessed by qRT-PCR and **C.** Western blot analysis of the corresponding protein levels of MYC, SP1, GATA2, RUNX1, CIP2A and SET. *β*-Actin and Calnexin were used as loading controls. Numbers indicate the protein quantification relative to *β*-Actin or Calnexin and assessed using Image J software (NIH, USA). **D.** Cell proliferation rates and **E.** PP2A activity levels with paired Western blot results of the amount of PP2A immunoprecipitated in each condition. Values are the mean ± SD of three independent experiments. *P < 0.05, **P < 0.01, ***P < 0.001, Students t-test analysis.

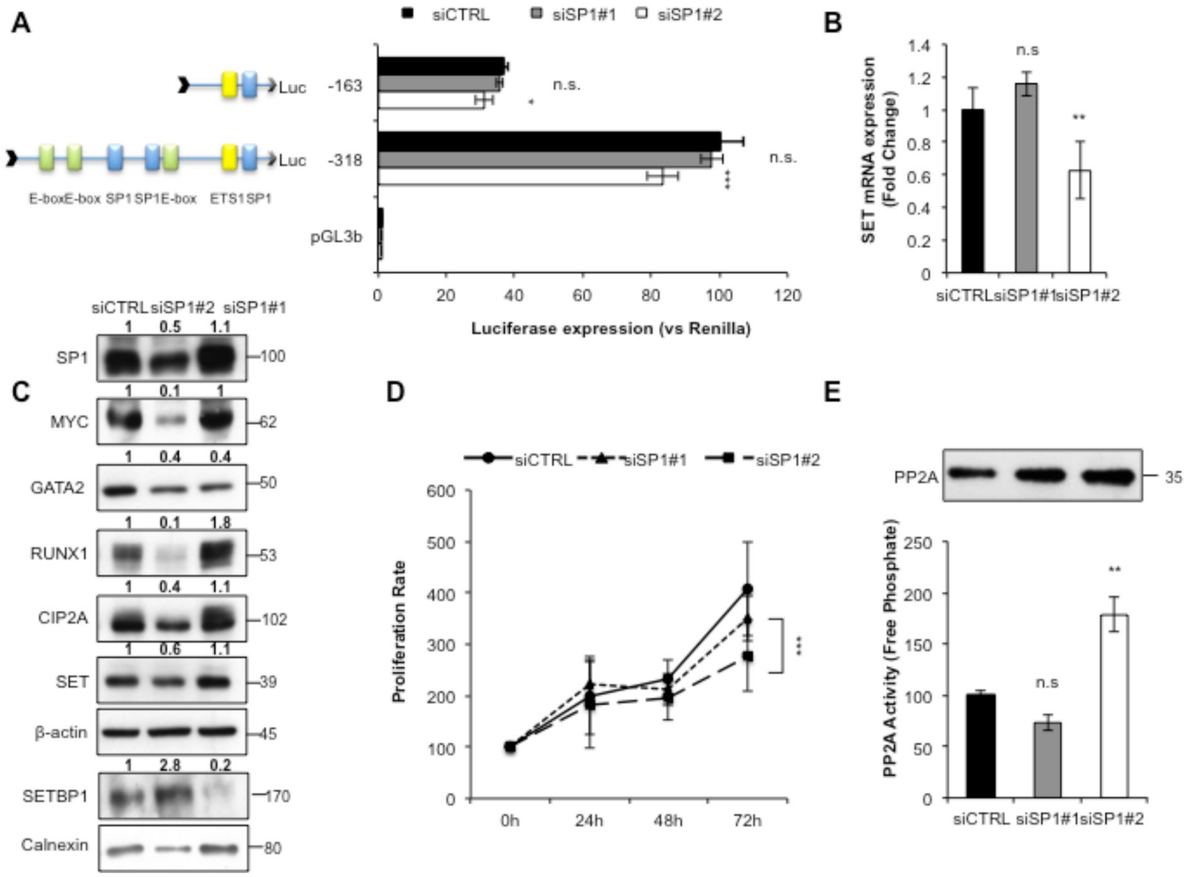

Supplementary Figure S9: SP1 activates SET transcription in AML. A. Luciferase assay in HEL cells transfected with two different siRNAs, siRNA#1 and siRNA#2. **B.** *SET* mRNA expression assessed by qRT-PCR and **C.** Western blot analysis of the corresponding protein levels of SP1, MYC, GATA2, RUNX1, CIP2A and SET. β-Actin and Calnexin were used as loading controls. Numbers indicate the protein quantification relative to β-Actin or Calnexin and assessed using Image J software (NIH, USA). **D.** Cell proliferation rates and **E.** PP2A activity levels with paired Western blot results of the amount of PP2A immunoprecipitated in each condition. Values are the mean ± SD of three independent experiments. *P < 0.05, **P < 0.01, ***P < 0.001, Students t-test analysis.

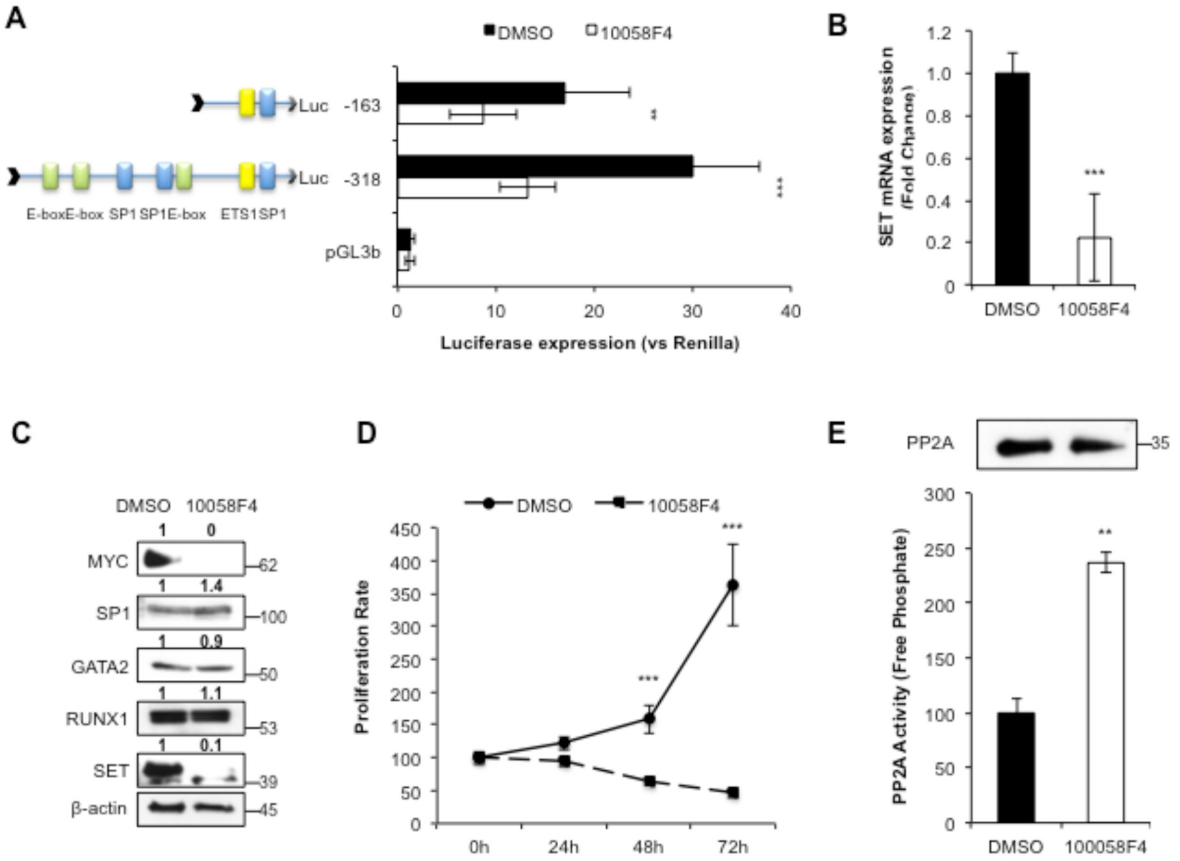

Supplementary Figure S10: MYC inhibition with 10058-F4 reduces SET expression and re-activates PP2A function in AML. A. Luciferase assay in HL-60 cells treated with DMSO or MYC inhibitor 10058-F4 60mM during 24h. **B.** *SET* mRNA expression assessed by qRT-PCR and **C.** Western blot analysis of the corresponding protein levels of MYC, SP1, GATA2, RUNX1 and SET. β-Actin was used as loading control. Numbers indicate the protein quantification relative to β-Actin and assessed using Image J software (NIH, USA). **D.** Cell proliferation rates and **E.** PP2A activity levels with paired Western blot results of the amount of PP2A immunoprecipitated in each condition. Data are the means ± SD of three independent experiments. *P < 0.05, **P < 0.01, ***P < 0.001, Students t-test analysis.

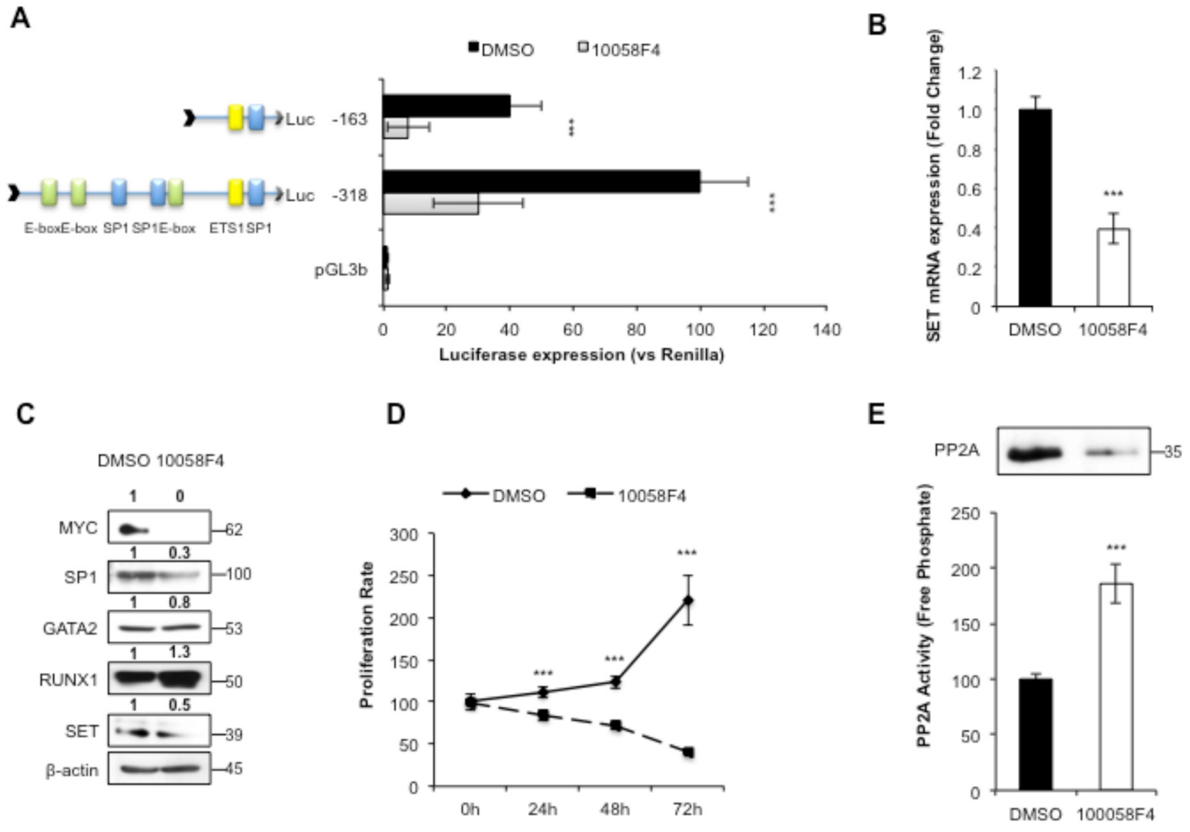

Supplementary Figure S11: MYC inhibition with 10058-F4 reduces SET expression and re-activates PP2A function in AML. A. Luciferase assay in HEL cells treated with DMSO or MYC inhibitor 10058-F4 60µM during 24h. **B.** *SET* mRNA expression assessed by qRT-PCR and **C.** Western blot analysis of the corresponding protein levels of MYC, SP1, GATA2, RUNX1 and SET. *β*-Actin was used as loading control. Numbers indicate the protein quantification relative to *β*-Actin and assessed using Image J software (NIH, USA). **D.** Cell proliferation rates and **E.** PP2A activity levels with paired Western blot results of the amount of PP2A immunoprecipitated in each condition. Values are the mean ± SD of three independent experiments *P < 0.05, **P < 0.01, ***P < 0.001, Students t-test analysis.

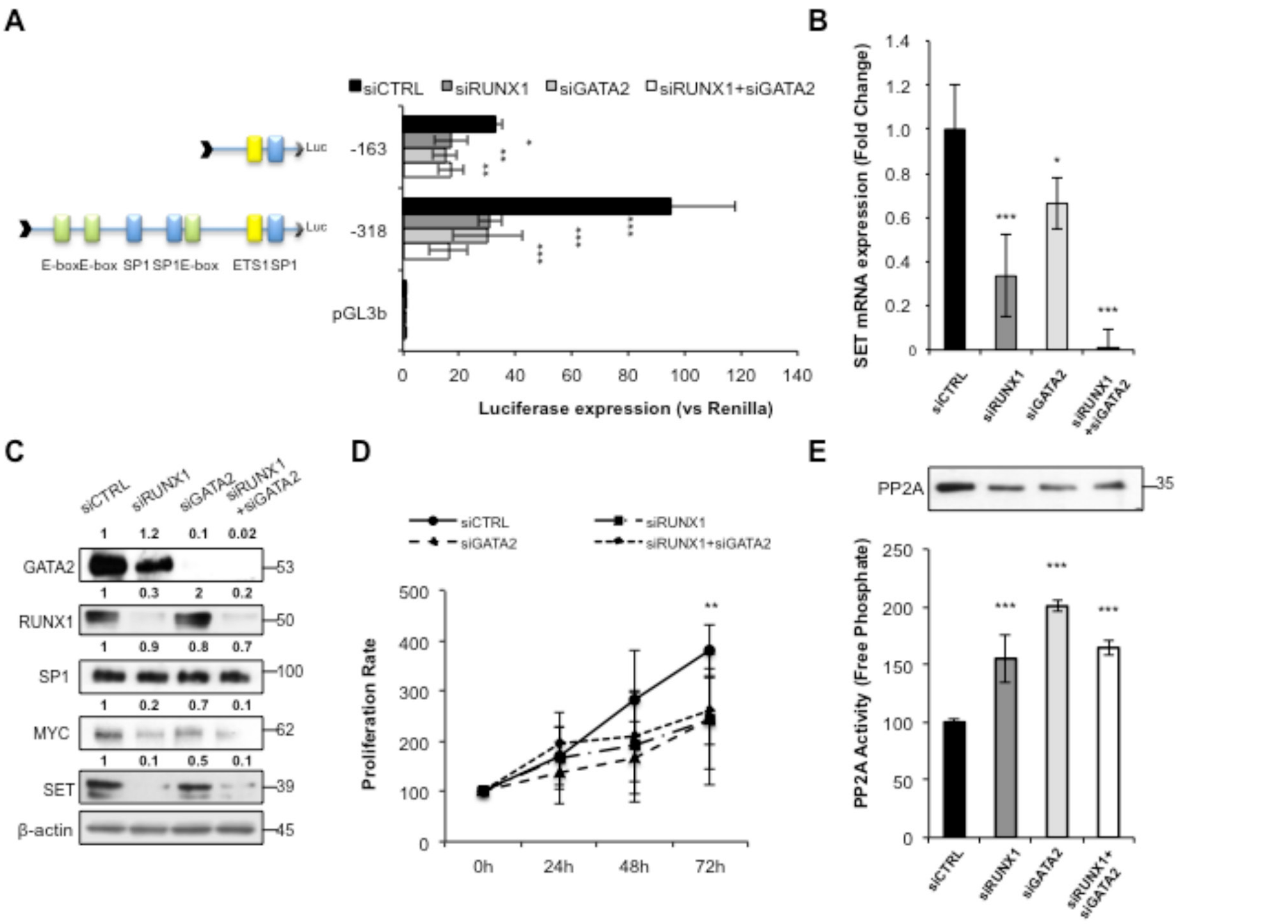

Supplementary Figure S12: RUNX1 and GATA2 are crucial for the activation of SET transcription and PP2A inactivation. A. Luciferase assay in HEL cells transfected with siRNA for RUNX1 and GATA2, alone or together. **B.** SET mRNA expression assessed by qRT-PCR and **C.** Western blot analysis with the corresponding RUNX1, GATA2, SP1, MYC and SET protein levels. *β*-Actin was used as loading control. Numbers indicate the protein quantification relative to β-Actin and assessed using Image J software (NIH, USA). **D.** Graphic representation of cell proliferation curve and **E.** PP2A activity with the corresponding Western blot of the amount of PP2A immunoprecipitated in each condition. Values are the mean ± SD of three independent experiments. Statistically significant differences are indicated: *P < 0.05, **P < 0.01, ***P < 0.001, One-way ANOVA with Bonferroni post-hoc test and Students t-test analysis were performed.

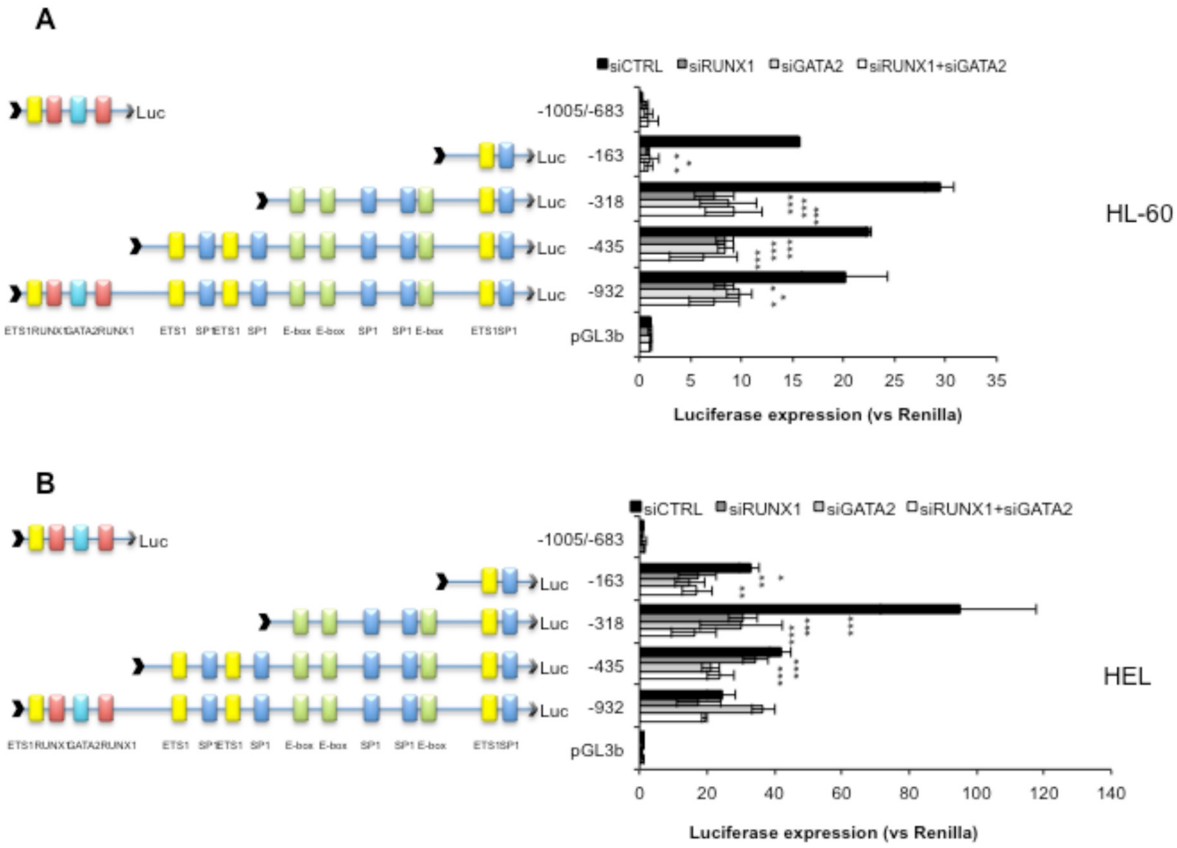

Supplementary Figure S13: Effect of RUNX1 and/or GATA2 depletion on SET promoter constructs. Extended data of the luciferase assay in HL-60 **A.** and HEL **B.** cells transfected with siRNA for RUNX1 and GATA2, alone or together, including the results of the -932bp and -435bp regions. Values are the mean ± SD of three independent experiments. Statistically significant differences are indicated: $*P < 0.05$, $**P < 0.01$, $***P < 0.001$, One-way ANOVA with Bonferroni post-hoc test and Students t-test analysis were performed.

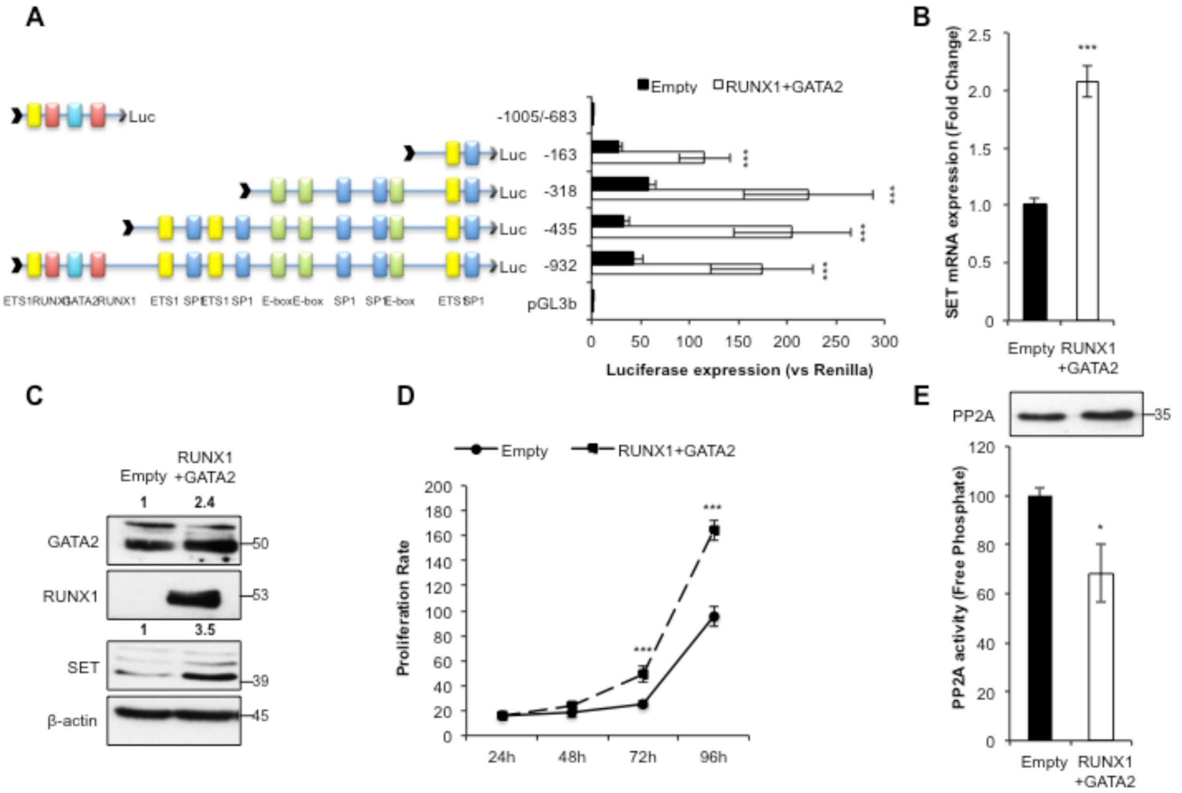

Supplementary Figure S14: Overexpression of RUNX1 and GATA2 activates SET expression and inactivates PP2A. **A.** Luciferase assay in HEK293t cells transfected with expression plasmids for RUNX1 and GATA2, together. **B.** SET expression by qRT-PCR and **C.** Western blot of the levels of RUNX1, GATA2 and SET. β-Actin was used as loading control. Numbers indicate the protein quantification relative to β-Actin and assessed using Image J software (NIH, USA). **D.** Cell proliferation curve and **E.** PP2A activity in cells with the simultaneous over-expression of RUNX1 and GATA2 compared to control (empty vector). The levels of PP2A activity are normalized to the amount of PP2A immunoprecitated in both conditions, represented by Western blot. Values are the mean ± SD of three independent experiments. Statistically significant differences are indicated: $*P < 0.05$, $**P < 0.01$, $***P < 0.001$, Students t-test analysis.

Supplementary Table S1: List of primers used.

See Supplementary File 1

Supplementary Table S2: List of antibodies used.

See Supplementary File 2
