# Supplementary figures and images for: MYC-dependent recruitment of RUNX1 and GATA2 on the SET oncogene promoter enhances PP2A inactivation in acute myeloid leukemia
Source: Oncotarget. 2016 Jun 6;8(33):53989–4003. doi: 10.18632/oncotarget.9840 (PMC5589557; doi:10.18632/oncotarget.9840)

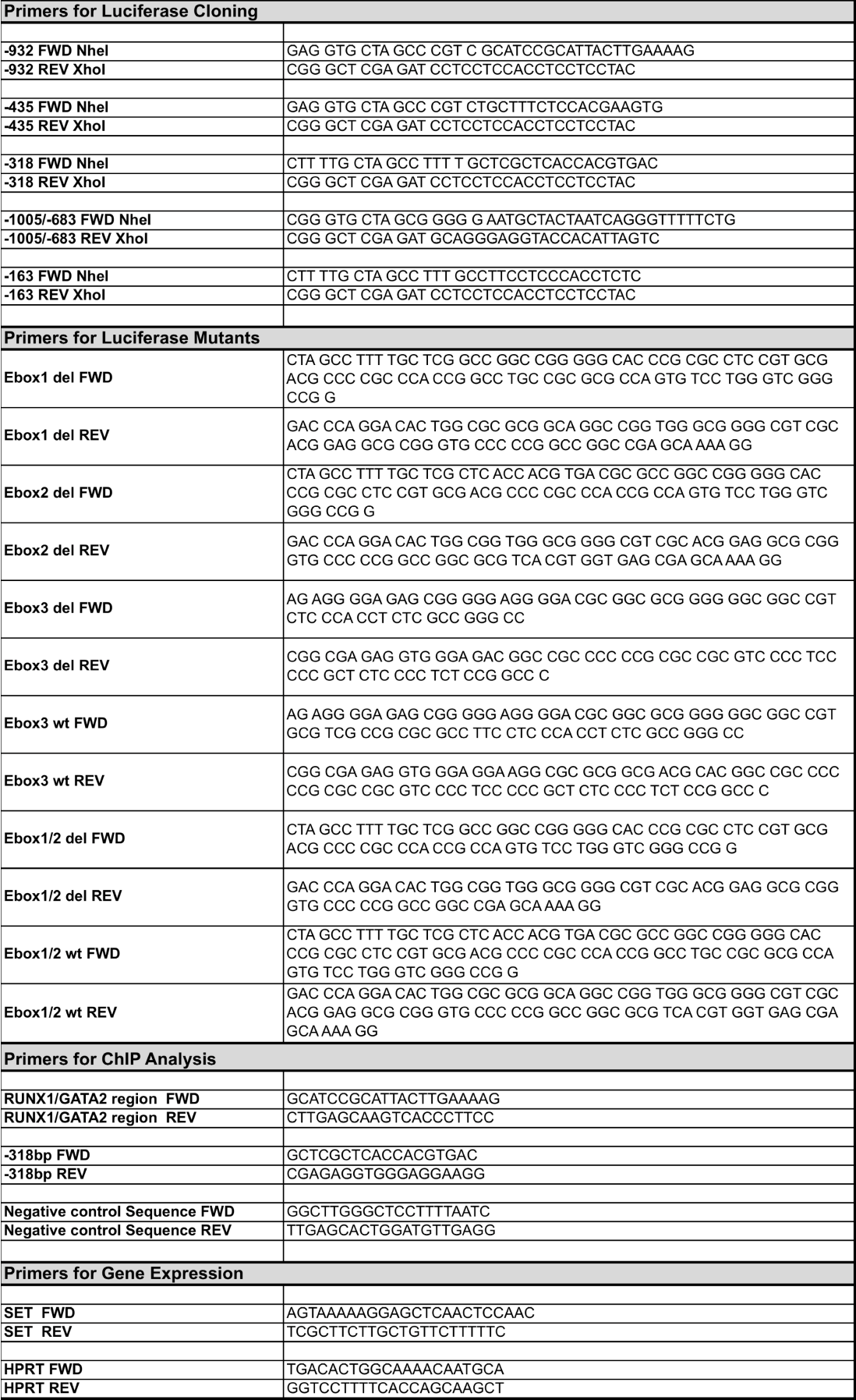

Supplement: Supplementary file 2 [file oncotarget-08-53989-s002.docx]

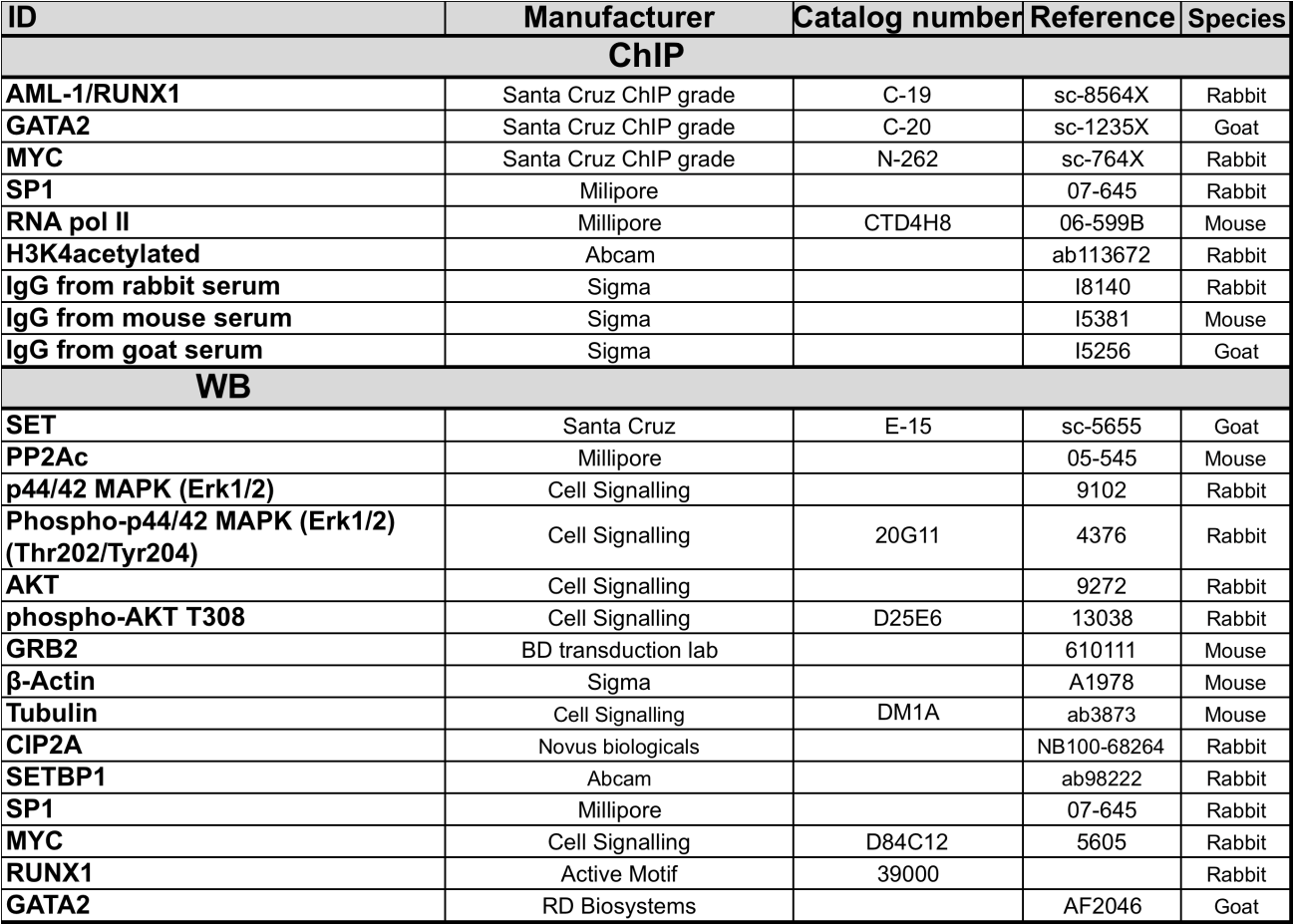

Supplement: Supplementary file 3 [file oncotarget-08-53989-s003.docx]
